# Supplementary material for: Loss of Trem2 in microglia leads to widespread disruption of cell coexpression networks in mouse brain
Source: Neurobiol Aging. 2018 Sep;69:151–66. doi: 10.1016/j.neurobiolaging.2018.04.019 (PMC6075941; doi:10.1016/j.neurobiolaging.2018.04.019)

Supplementary file 1:

- **PCA plot showing samples clustering by age and by tissue**

- **4 months cortex WT vs KO comparison: MAplot, qqplot , variance and volcano plots**


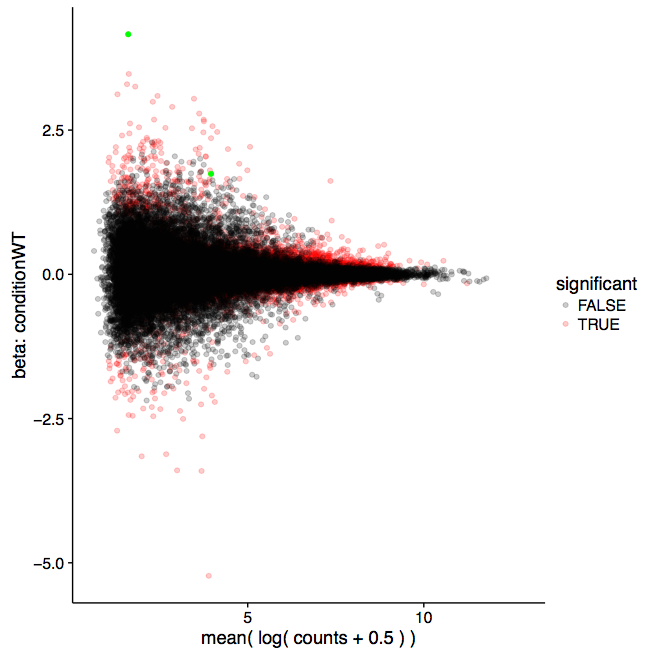

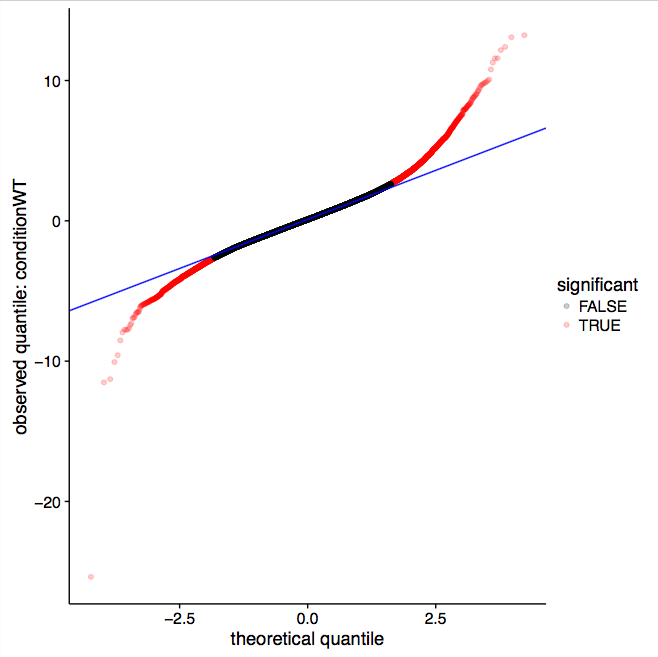

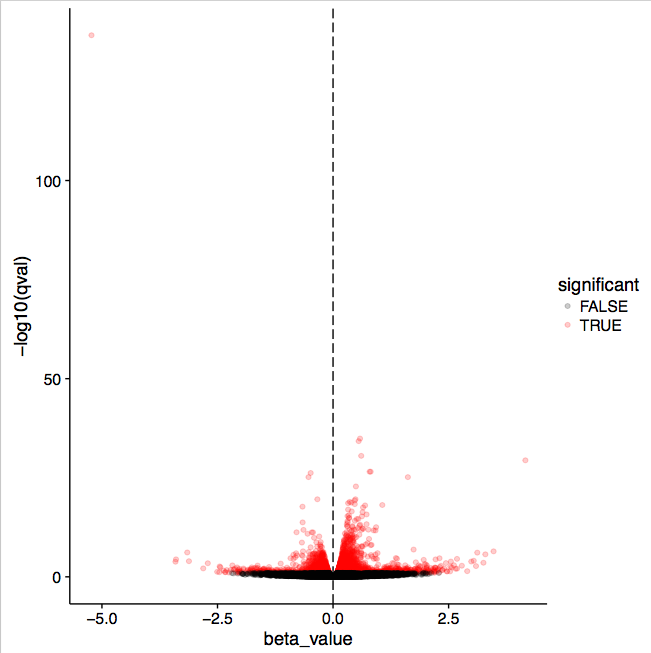


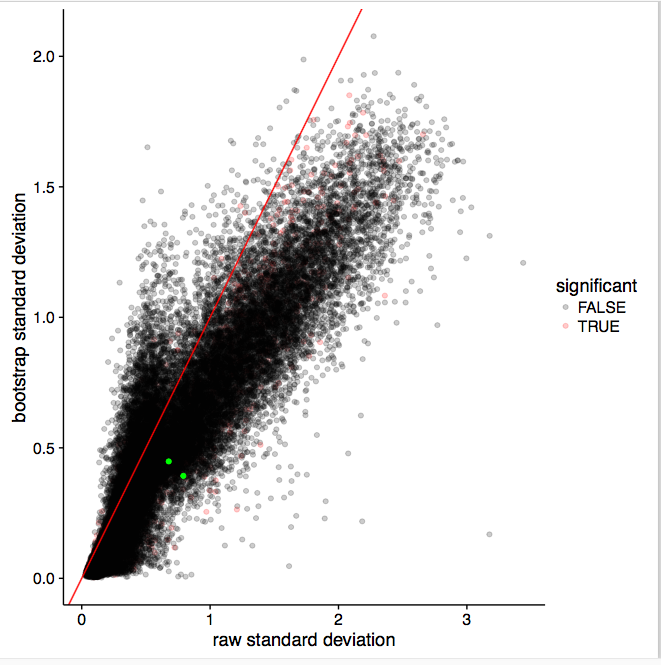


- **4 months hippocampus WT vs KO comparison: MAplot, qqplot , variance and volcano plots**

**
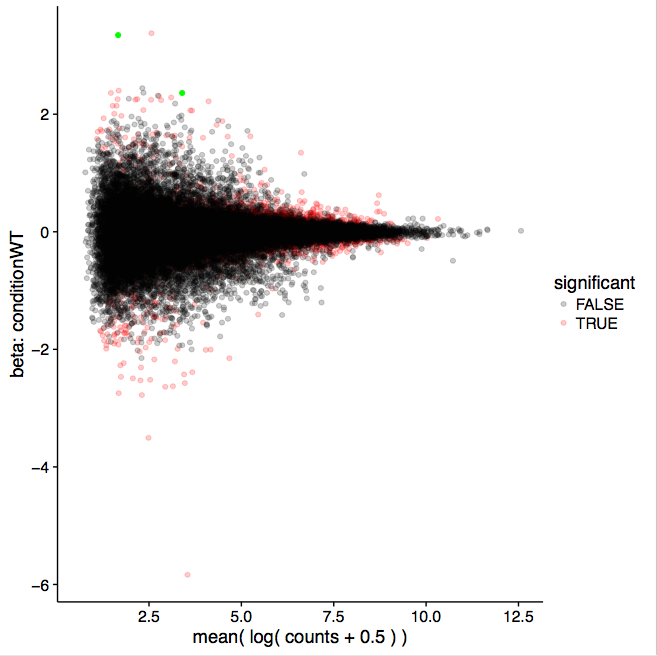

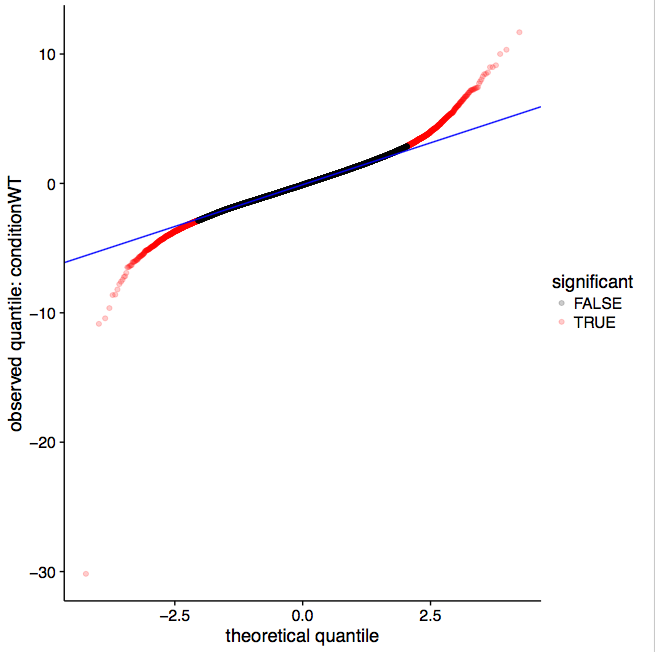

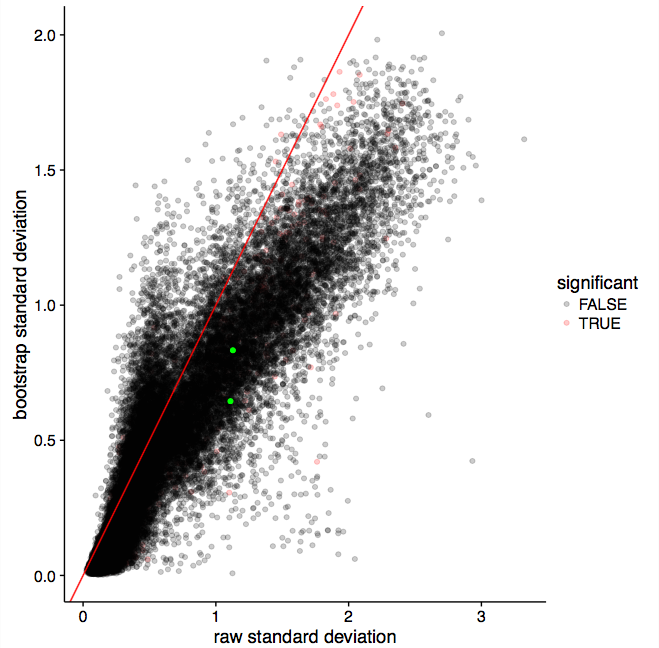

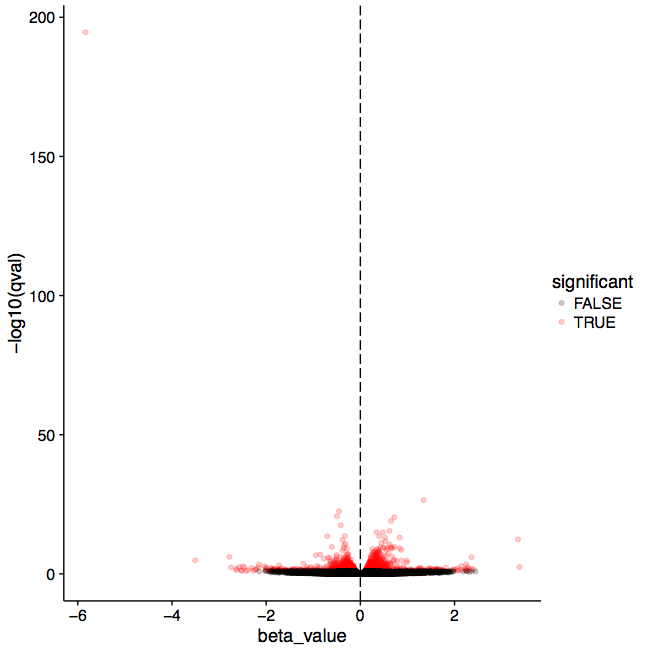
**

- **8 months cortex WT vs KO comparison: MAplot, qqplot , variance and volcano plots**

**
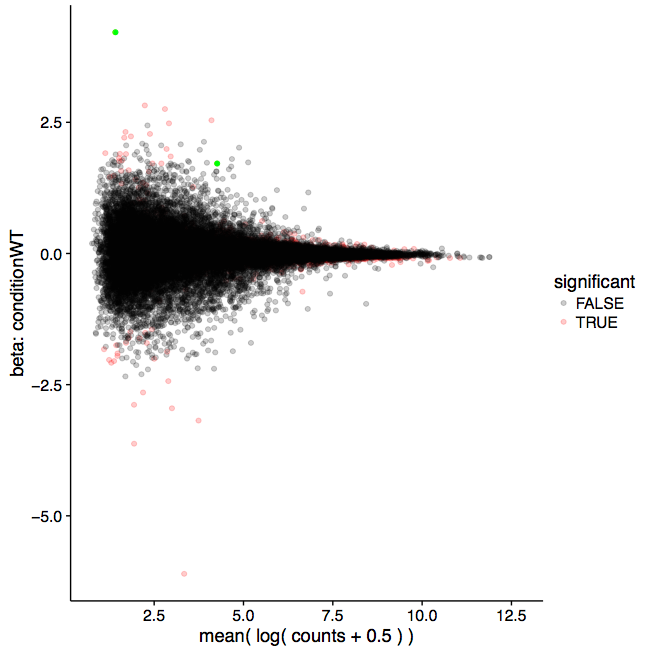

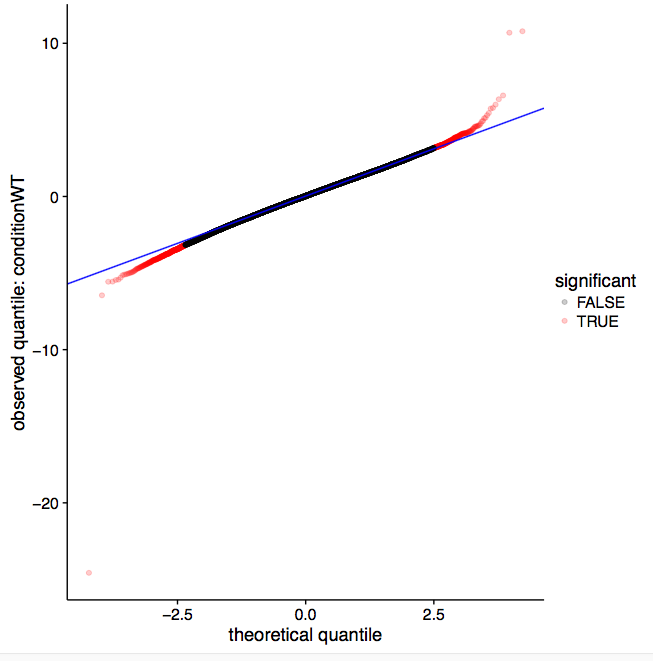

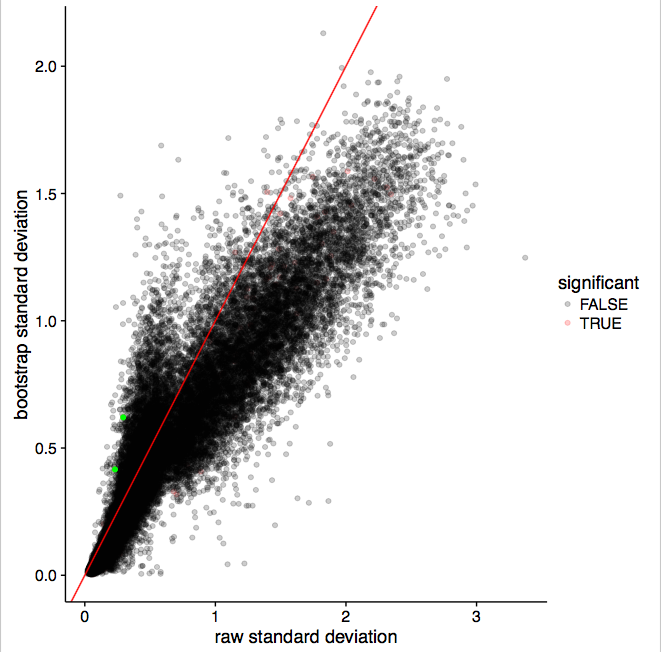

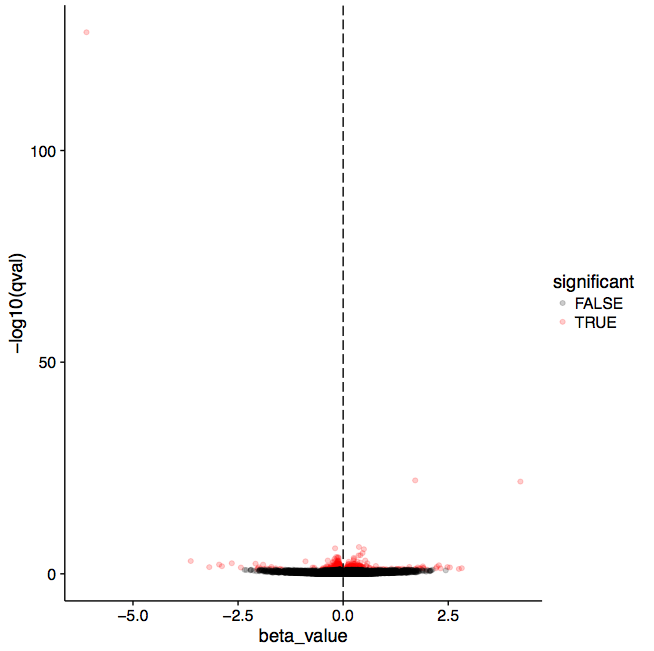
**

- **8 months hippocampus WT vs KO comparison: MAplot, qqplot , variance and volcano plots**


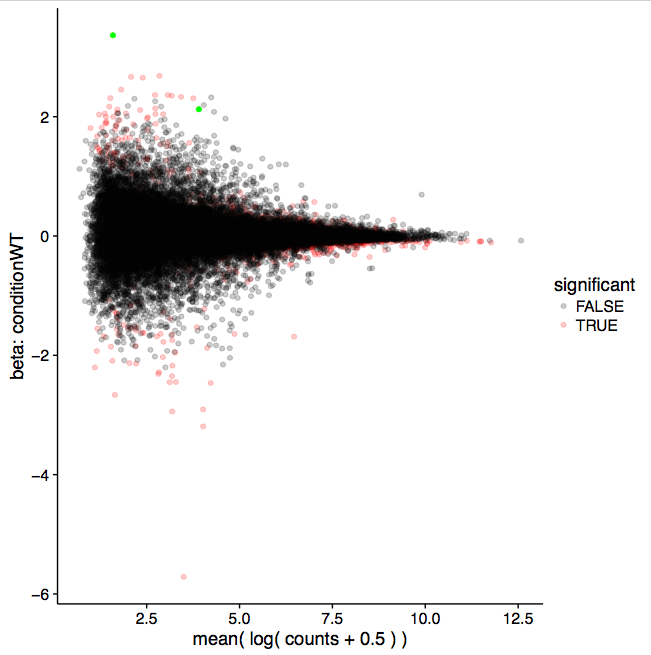

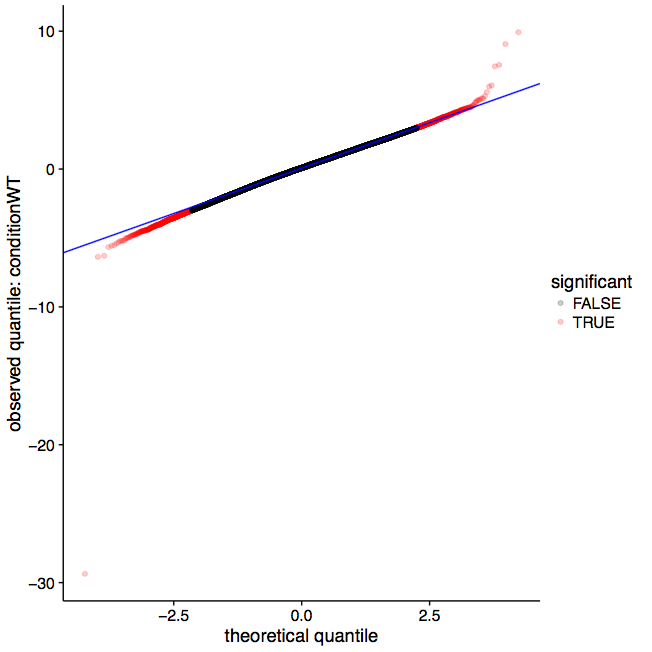

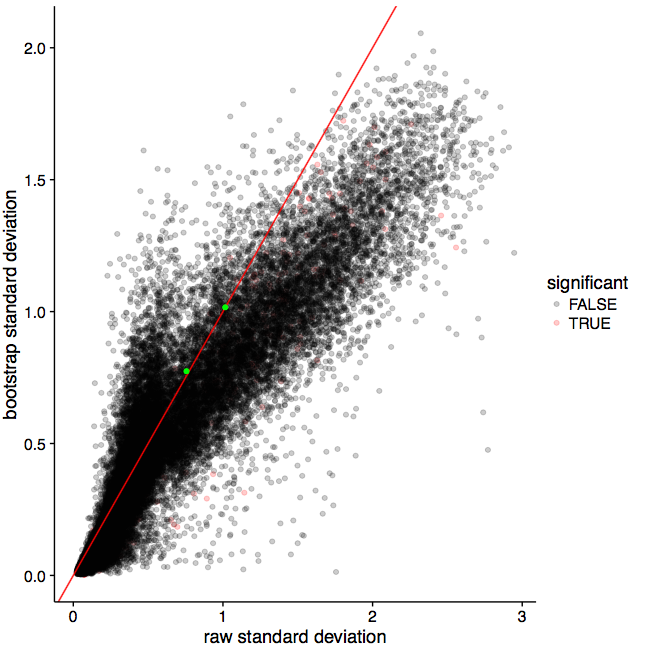

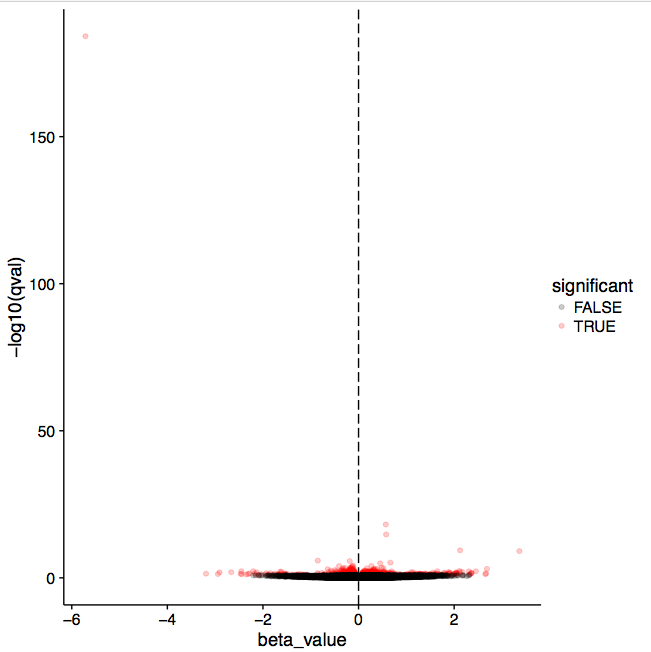

Supplement: S1 File V2 [file mmc2.docx]
