# Supplementary material for: Loss of Trem2 in microglia leads to widespread disruption of cell coexpression networks in mouse brain
Source: Neurobiol Aging. 2018 Sep;69:151–66. doi: 10.1016/j.neurobiolaging.2018.04.019 (PMC6075941; doi:10.1016/j.neurobiolaging.2018.04.019)
Supplement: S2 File V2 [file mmc3.docx]

WT network samples clustering and scale free topology fit

KO network samples clustering and scale free topology fit
