# Supplementary material for: Loss of Trem2 in microglia leads to widespread disruption of cell coexpression networks in mouse brain
Source: Neurobiol Aging. 2018 Sep;69:151–66. doi: 10.1016/j.neurobiolaging.2018.04.019 (PMC6075941; doi:10.1016/j.neurobiolaging.2018.04.019)

Venn diagram of differential expression gene overlap. The supplementary file 6 can be visualized uploading it to <http://www.interactivenn.net> and details about the overlapping genes can be obtained by clicking on every overlapping section of the diagram


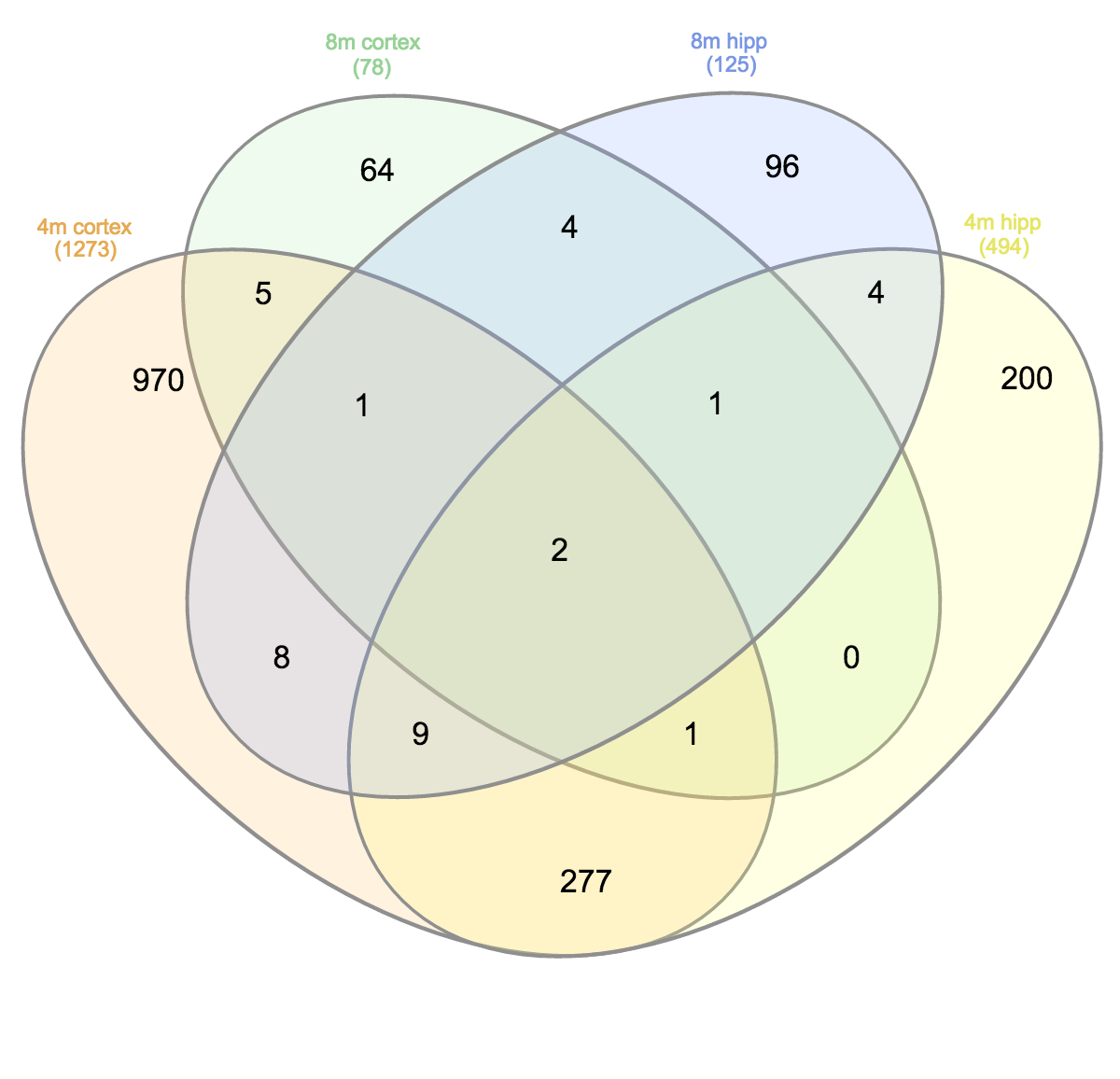

Supplement: S5 File [file mmc6.docx]
