# Supplementary material for: Loss of Trem2 in microglia leads to widespread disruption of cell coexpression networks in mouse brain
Source: Neurobiol Aging. 2018 Sep;69:151–66. doi: 10.1016/j.neurobiolaging.2018.04.019 (PMC6075941; doi:10.1016/j.neurobiolaging.2018.04.019)
Supplement: Supplementary File 7 [file mmc8.pdf]

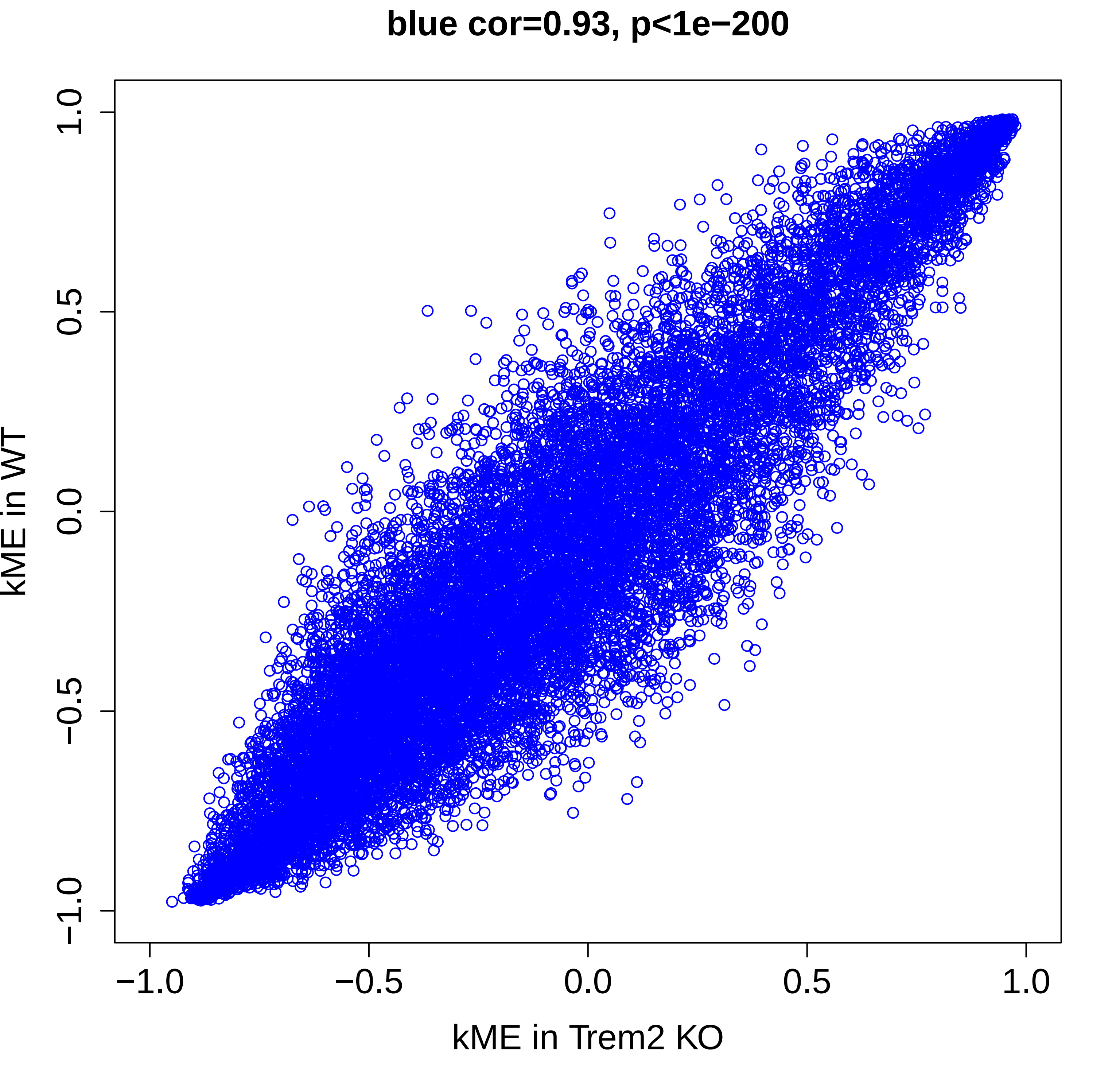
blue cor=0.93, p<1e−200
kME in WT
kME in Trem2 KO
−1.0
−0.5
0.0
0.5
1.0

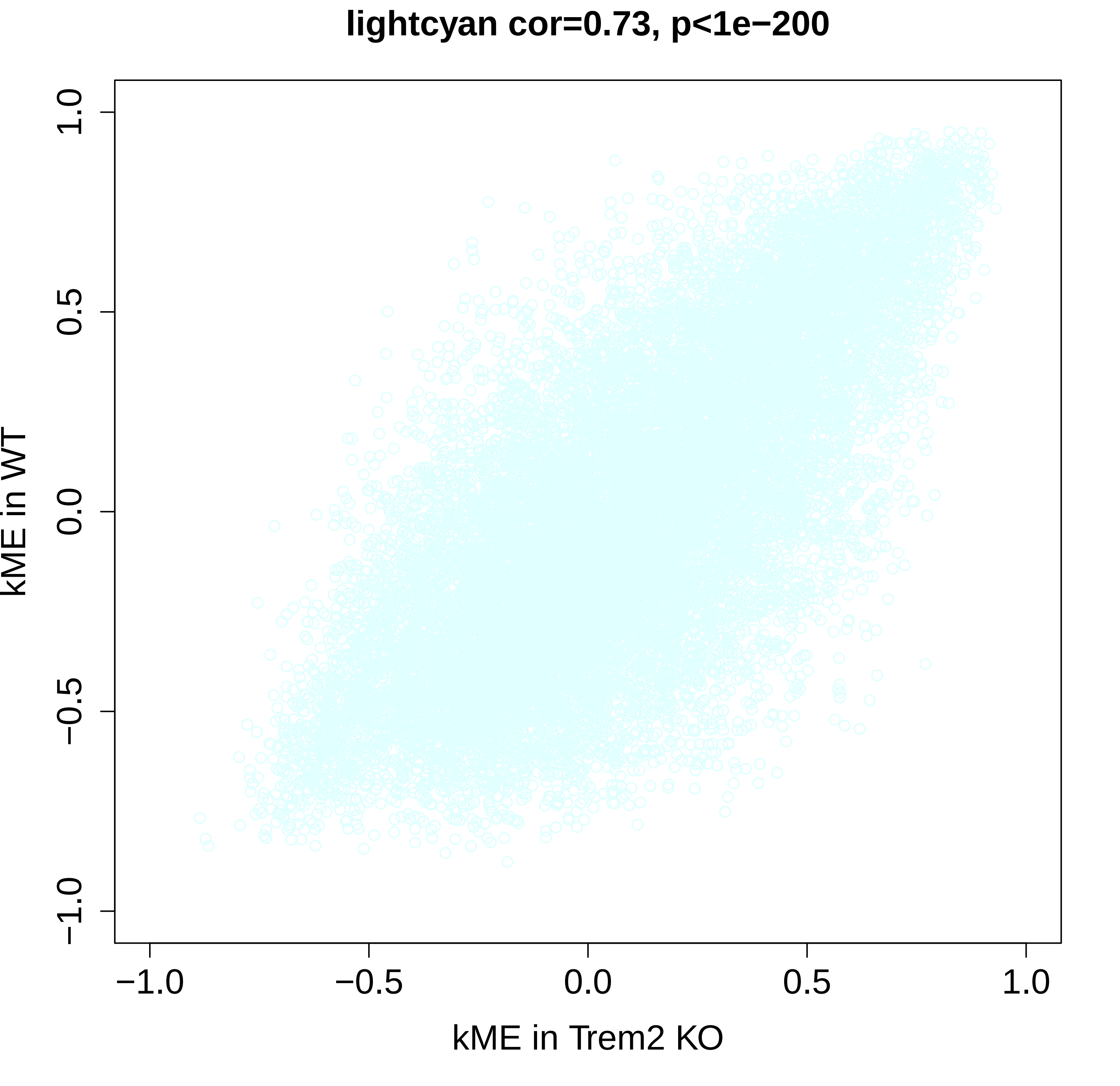
lightcyan cor=0.73, p<1e−200
kME in WT
kME in Trem2 KO
1.0
0.5
0.0
−0.5
−1.0
−1.0
−0.5
0.0
0.5
1.0

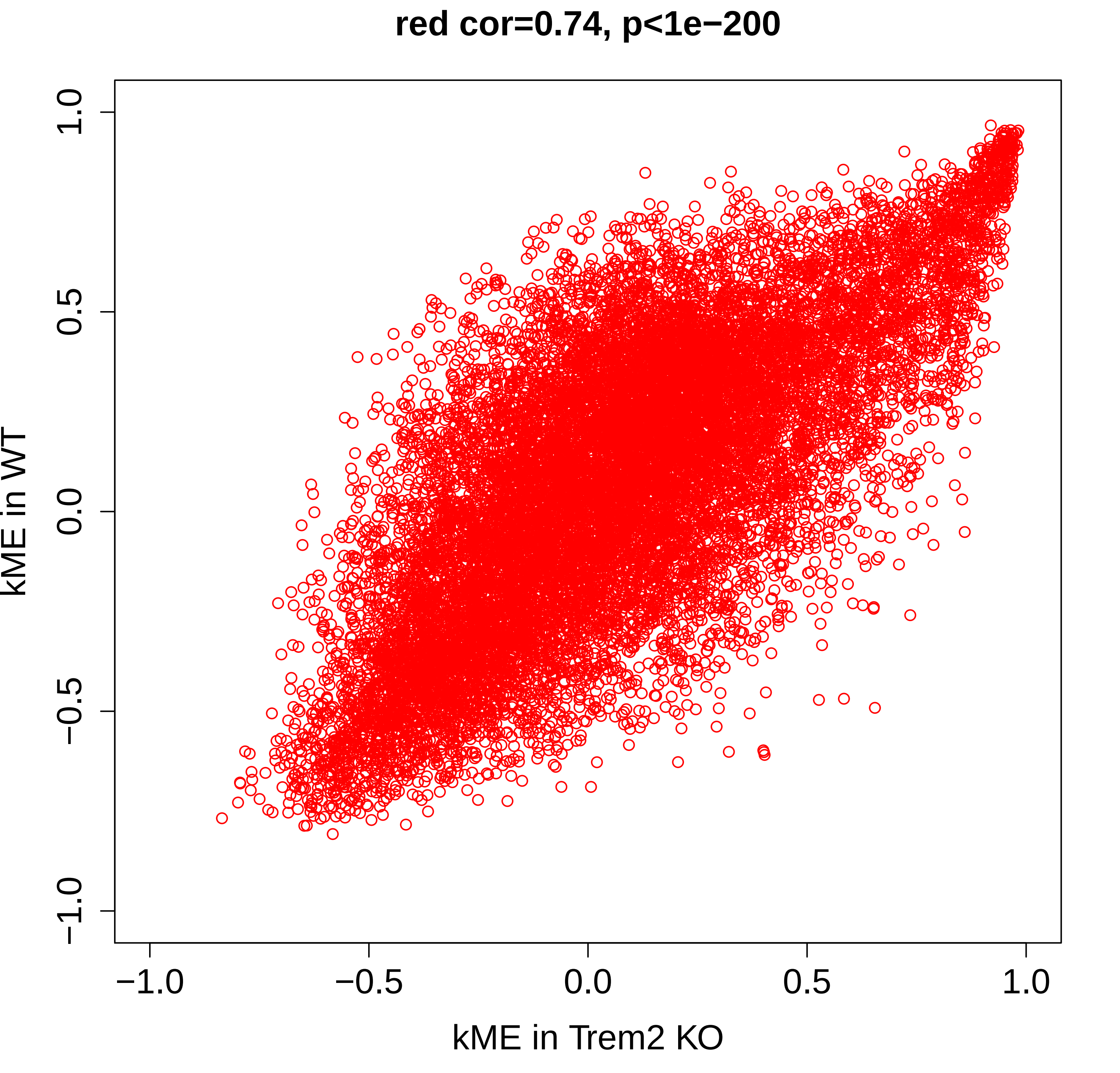
red cor=0.74, p<1e−200
kME in WT
1.0
0.5
0.0
−0.5
−1.0
−1.0
−0.5
0.0
0.5
1.0
kME in Trem2 KO

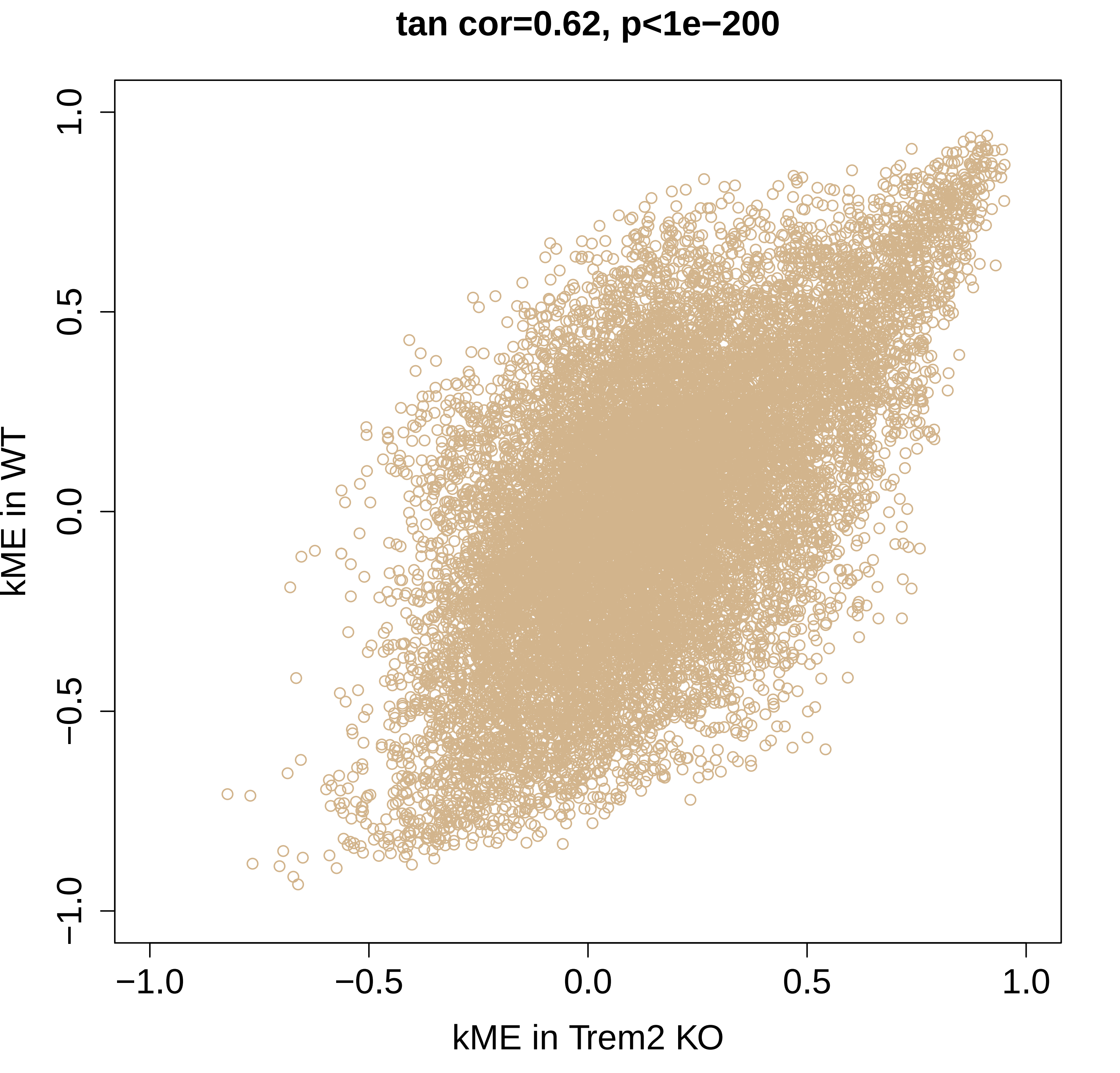

tan cor=0.62, p<1e−200
kME in WT
kME in Trem2 KO
1.0
0.5
0.0
−0.5
−1.0

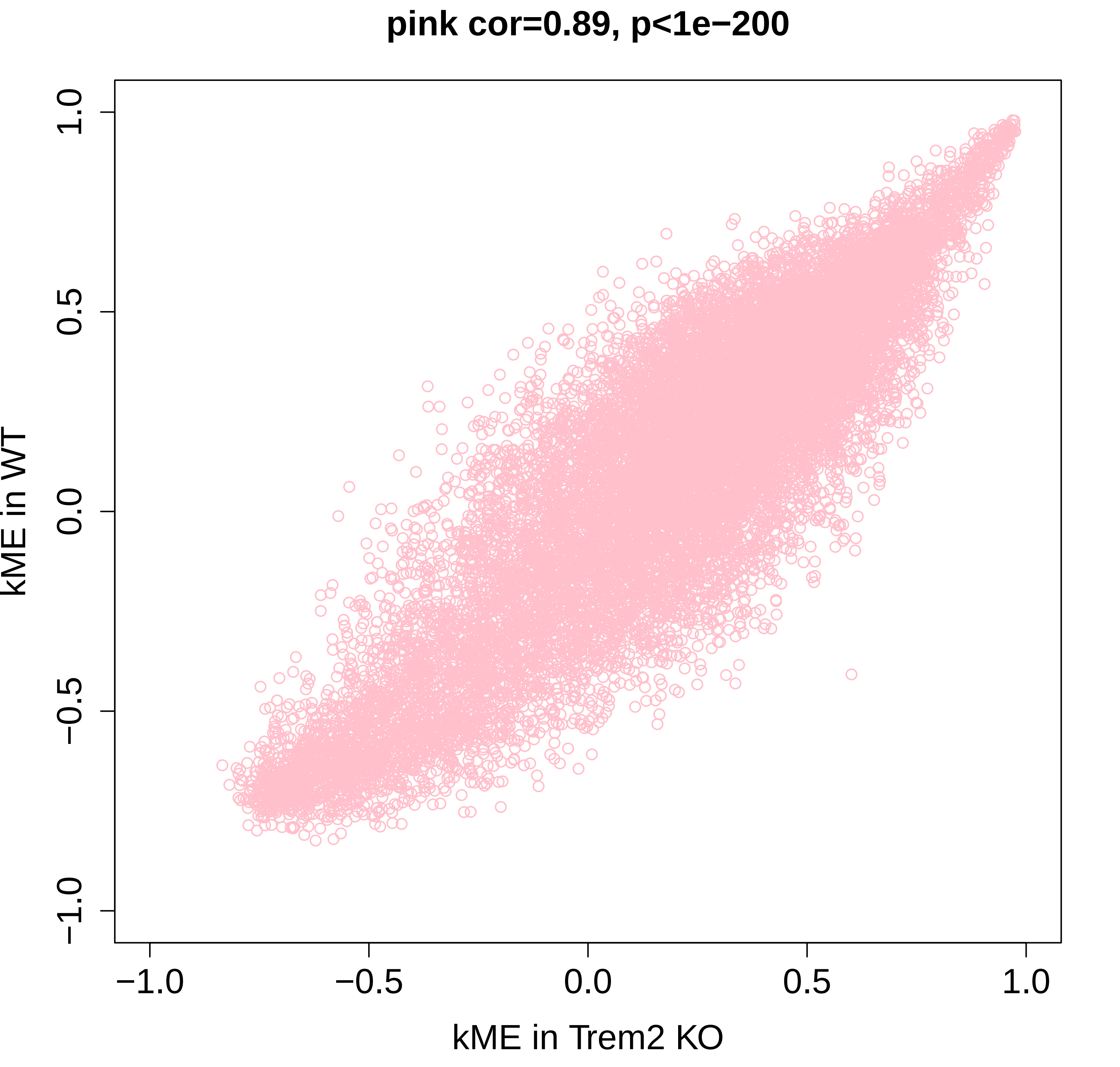
pink cor=0.89, p<1e−200
kME in WT
kME in Trem2 KO
1.0
0.5
0.0
−0.5
−1.0
−1.0
−0.5
0.0
0.5
1.0

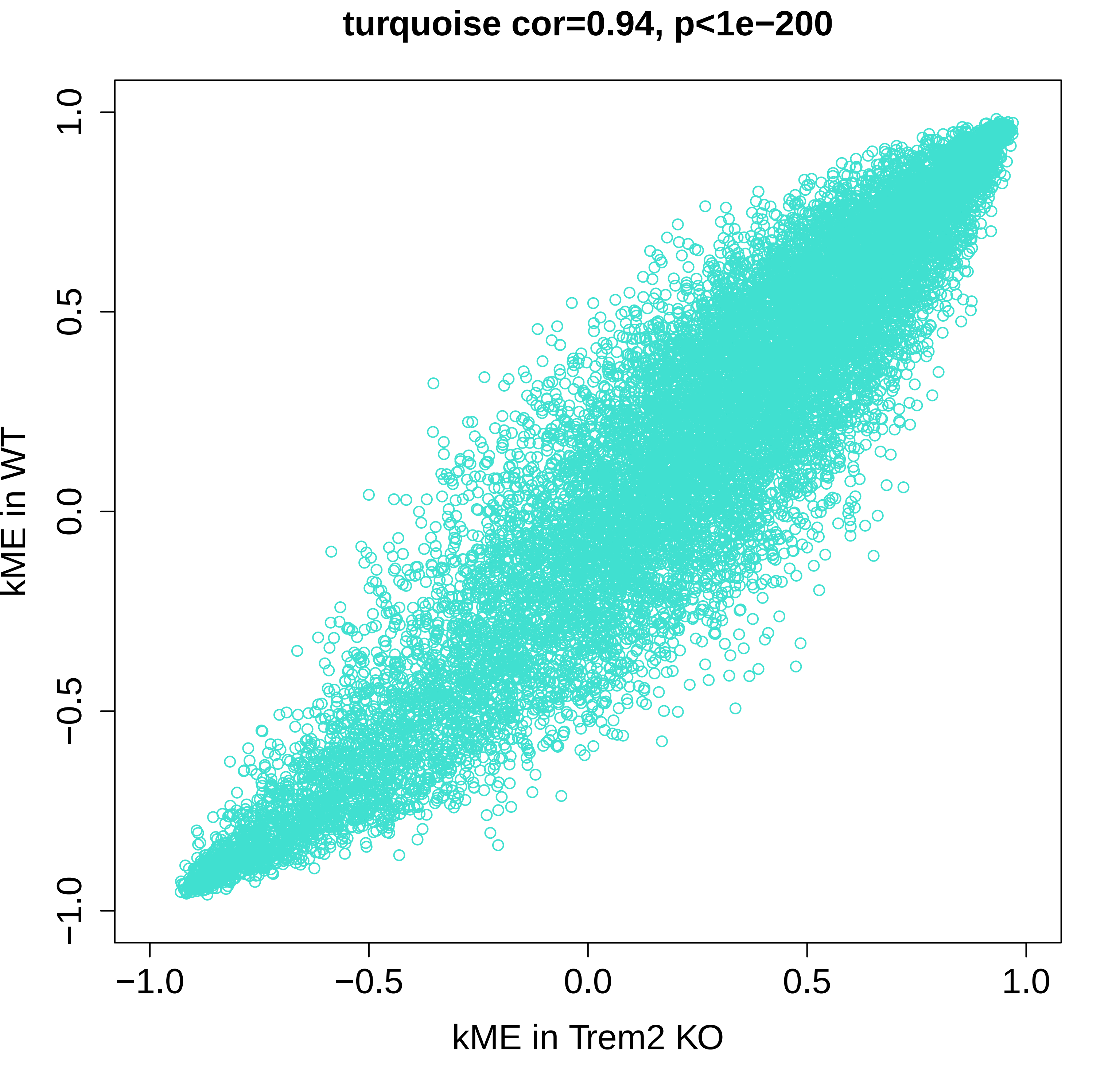
turquoise cor=0.94, p<1e−200
kME in WT
kME in Trem2 KO
1.0
0.5
0.0
−0.5
−1.0
−1.0
−0.5
0.0
0.5
1.0

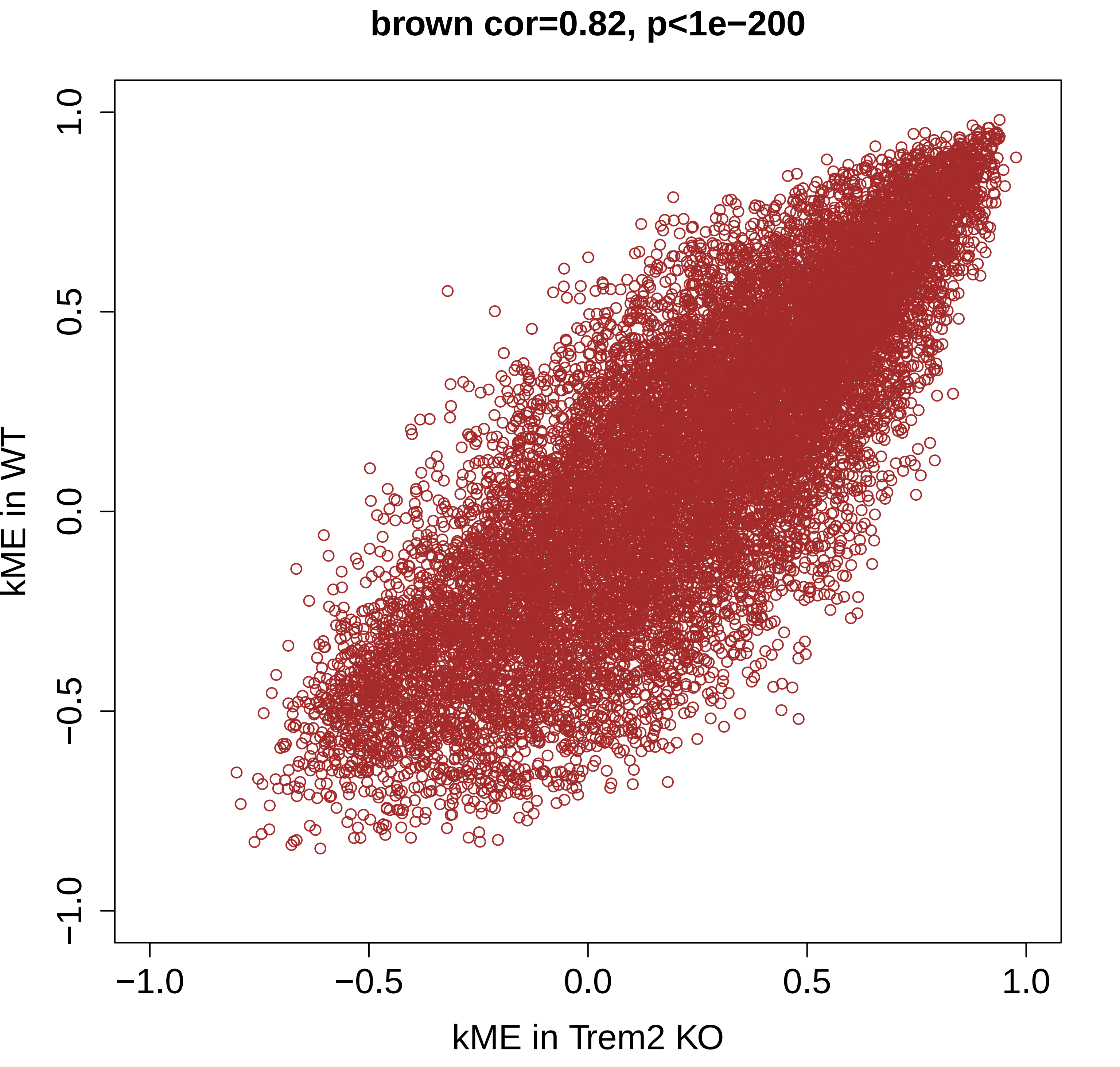
brown cor=0.82, p<1e−200
kME in WT
kME in Trem2 KO
1.0
0.5
0.0
−0.5
−1.0
−1.0
−0.5
0.0
0.5
1.0

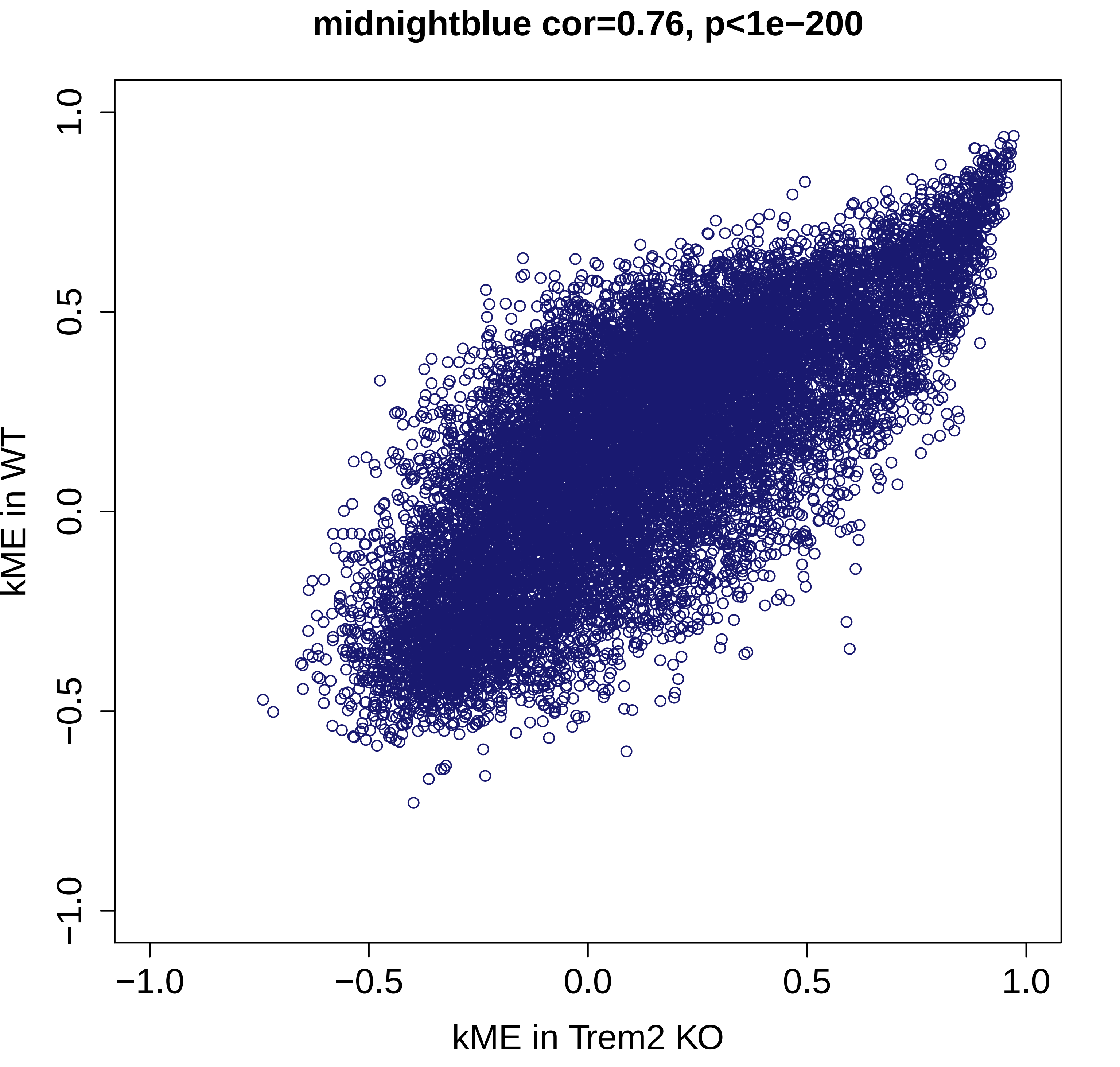
midnightblue cor=0.76, p<1e−200
kME in WT
kME in Trem2 KO
1.0
0.5
0.0
−0.5
−1.0
−1.0
−0.5
0.0
0.5
1.0
