# Supplementary material for: Loss of Trem2 in microglia leads to widespread disruption of cell coexpression networks in mouse brain
Source: Neurobiol Aging. 2018 Sep;69:151–66. doi: 10.1016/j.neurobiolaging.2018.04.019 (PMC6075941; doi:10.1016/j.neurobiolaging.2018.04.019)
Supplement: Supplementary File 8 [file mmc9.pdf]

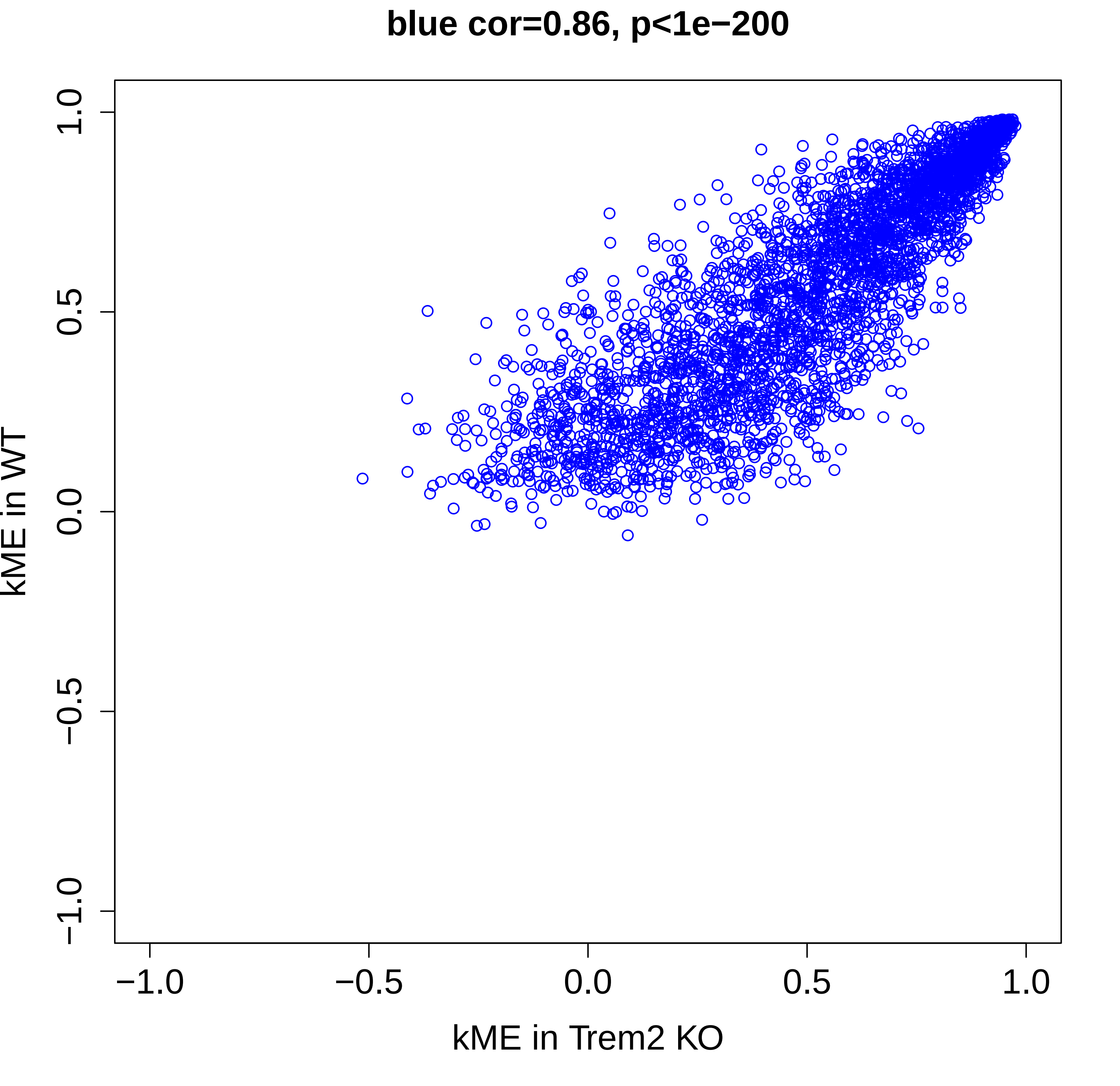
blue cor=0.86, p<1e−200
kME in WT
kME in Trem2 KO
1.0
0.5
0.0
−0.5
−1.0
−1.0
−0.5
0.0
0.5
1.0

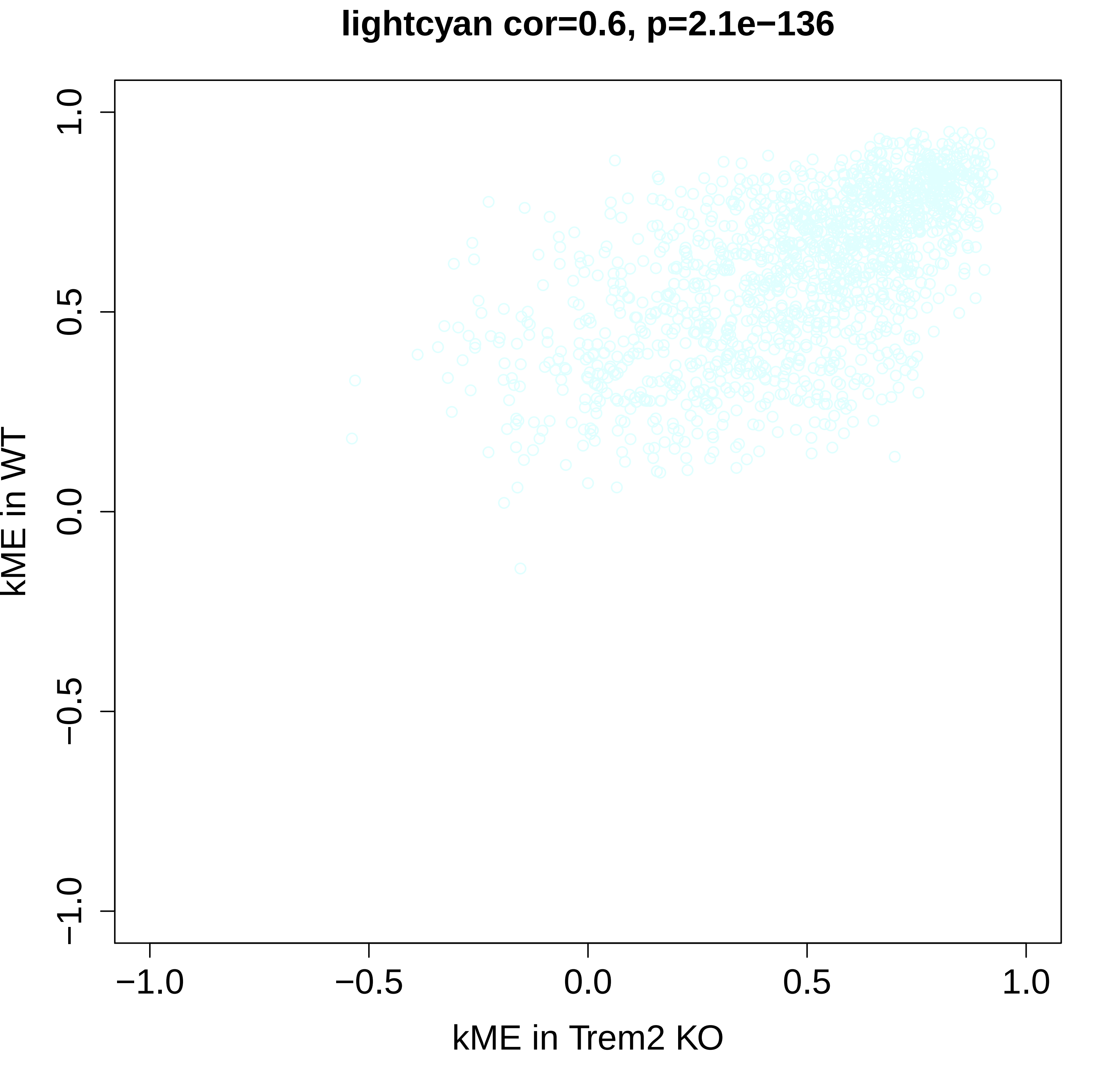

lightcyan cor=0.6, p=2.1e−136
kME in WT
kME in Trem2 KO
1.0
0.5
0.0
−0.5
−1.0
−1.0
−0.5
0.0
0.5
1.0

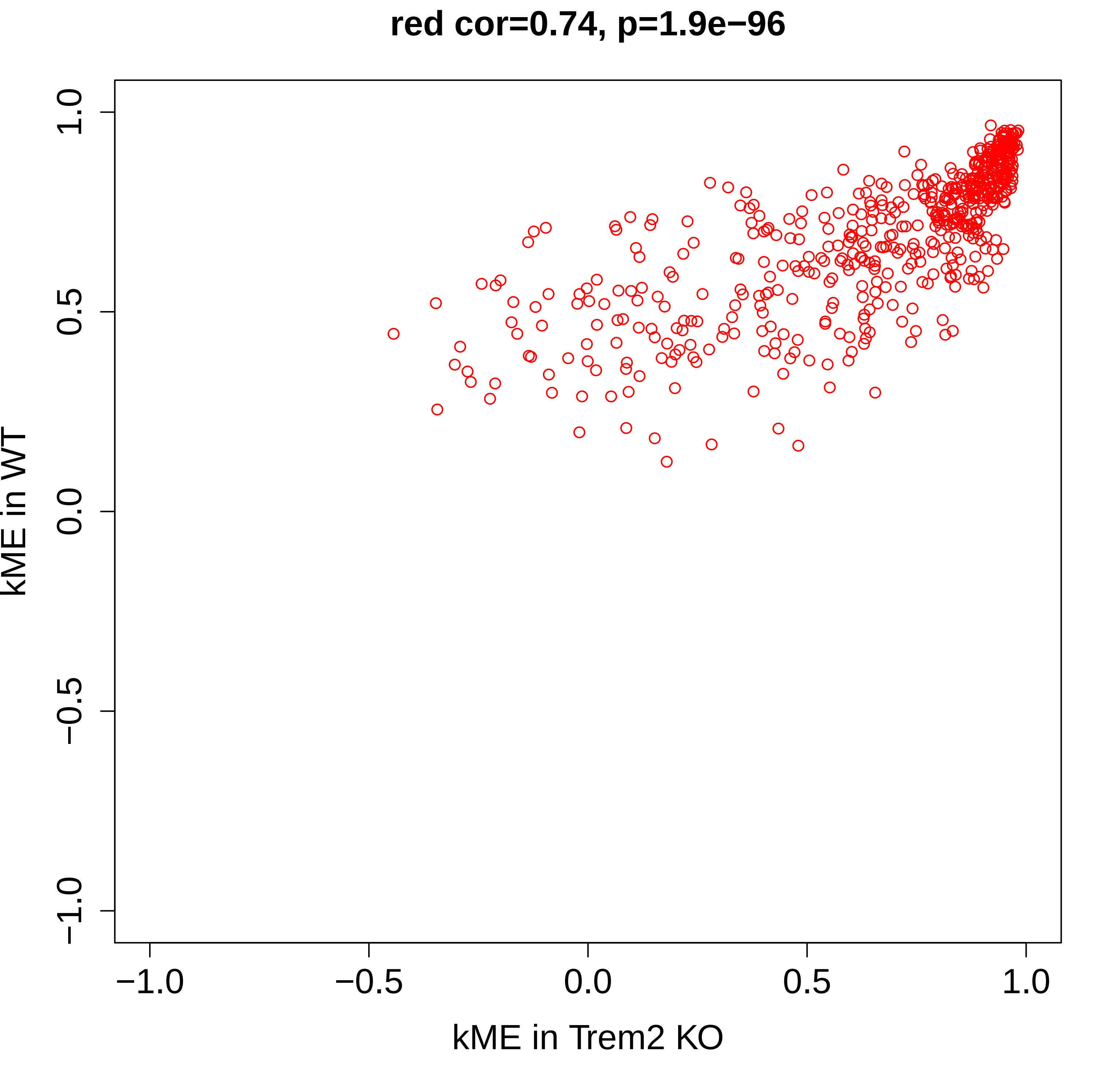
red cor=0.74, p=1.9e−96
kME in WT
kME in Trem2 KO
1.0
0.5
0.0
−0.5
−1.0
−1.0
−0.5
0.0
0.5
1.0

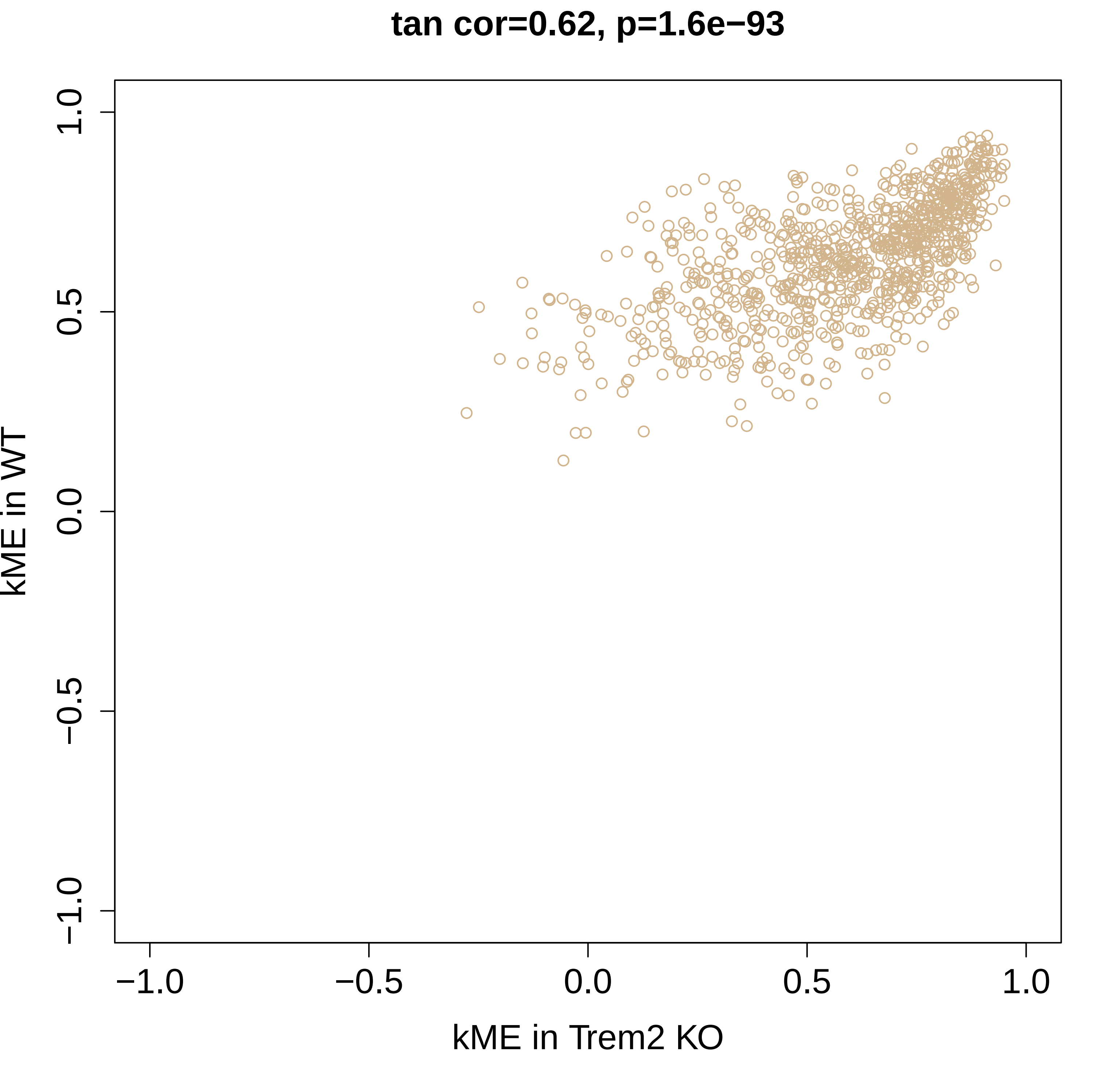
tan cor=0.62, p=1.6e−93
kME in WT
kME in Trem2 KO
1.0
0.5
0.0
−0.5
−1.0
−1.0
−0.5
0.0
0.5
1.0

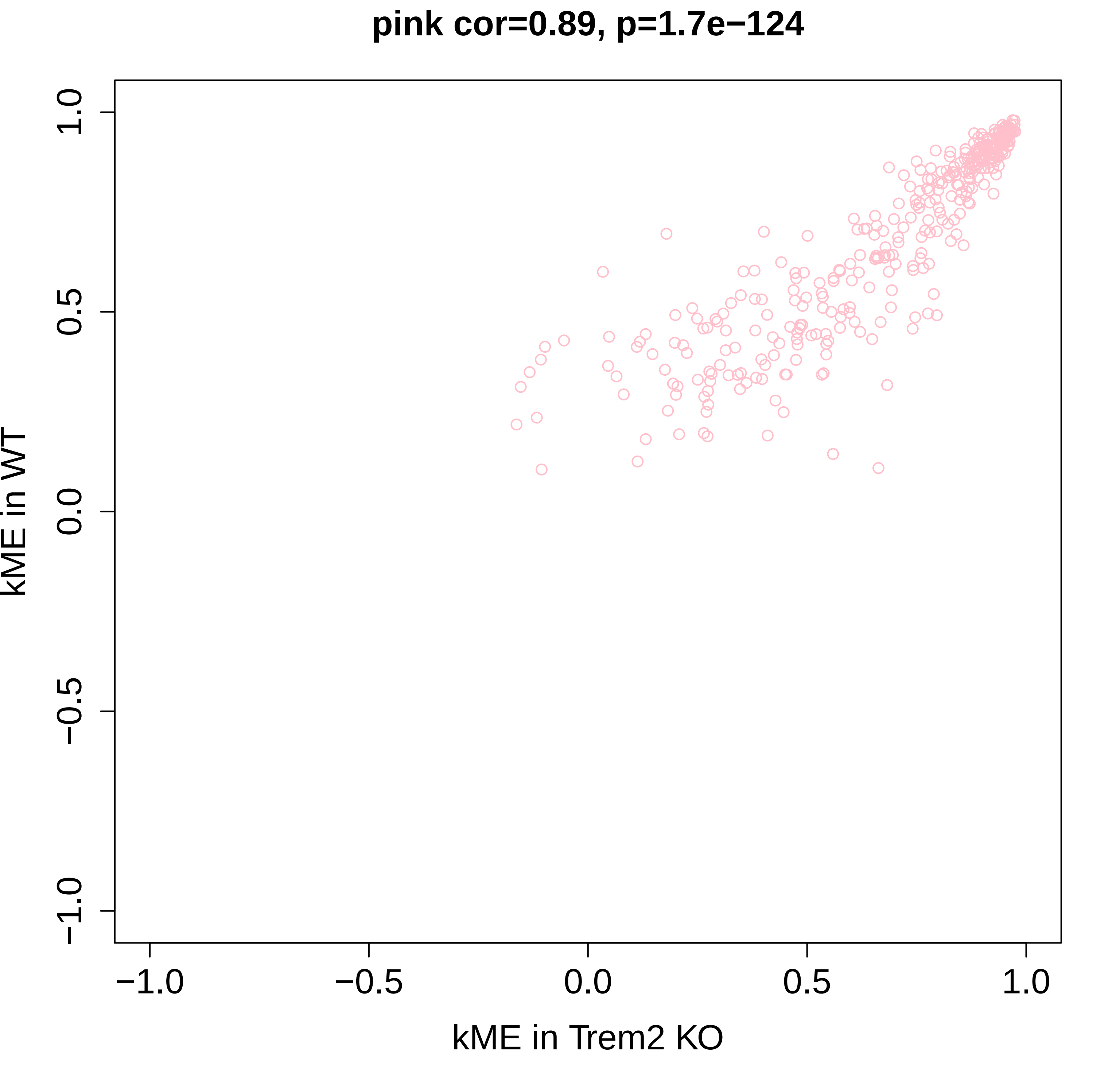
pink cor=0.89, p=1.7e−124
kME in WT
kME in Trem2 KO
1.0
0.5
0.0
−0.5
−1.0
−1.0
−0.5
0.0
0.5
1.0

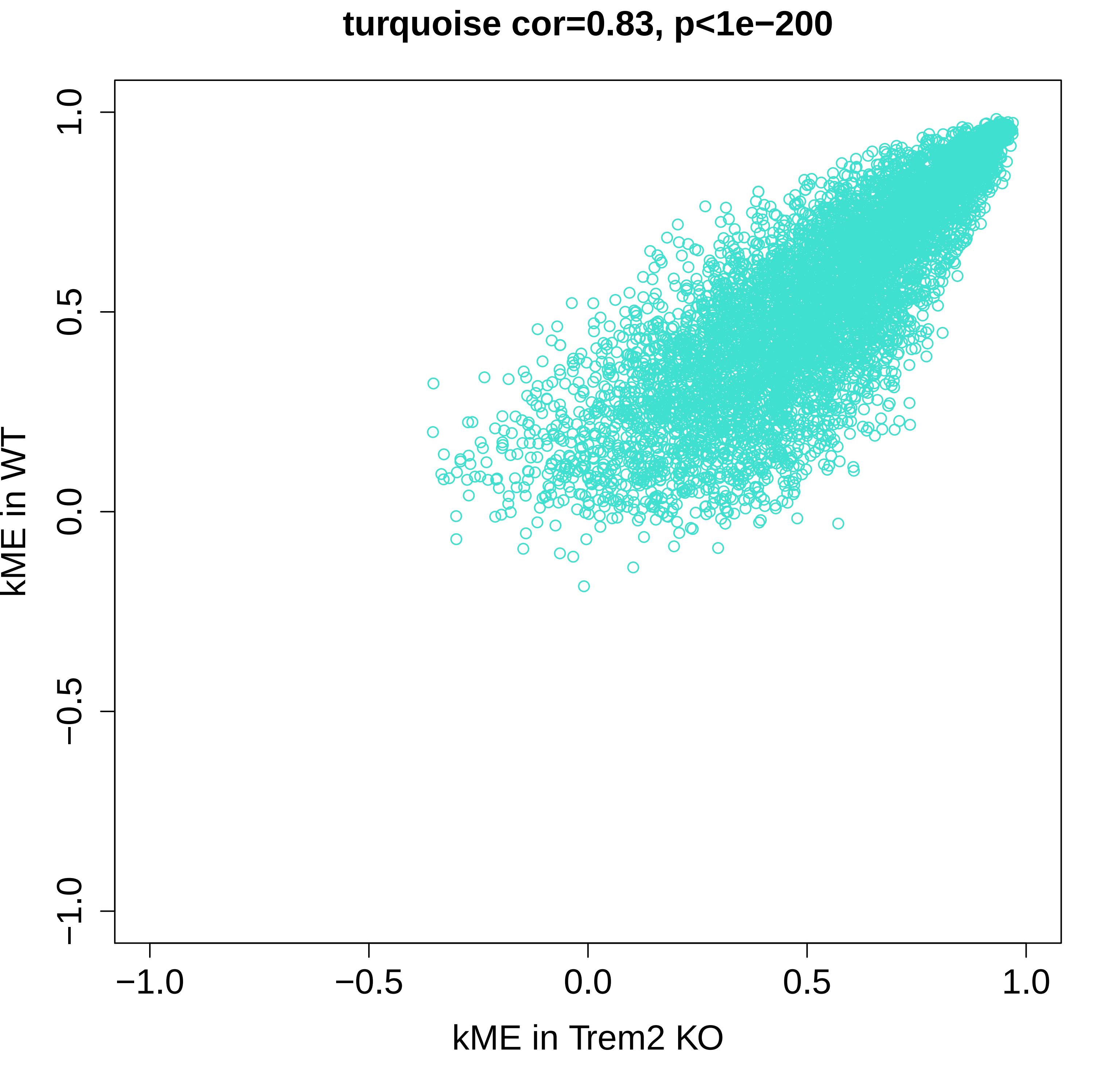

turquoise cor=0.83, p<1e−200
kME in WT
kME in Trem2 KO
1.0
0.5
0.0
−0.5
−1.0
−1.0
−0.5
0.0
0.5
1.0

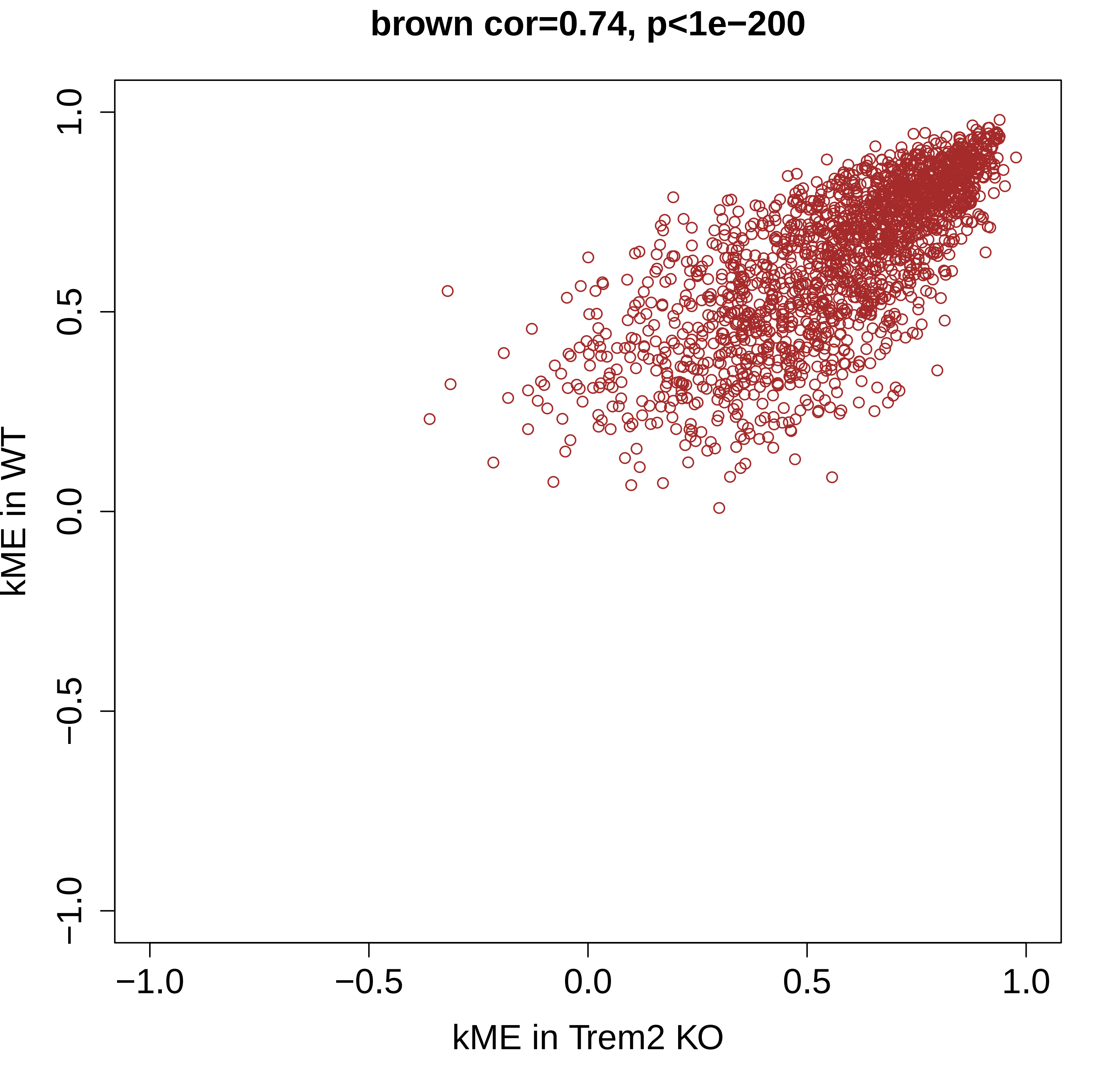

brown cor=0.74, p<1e−200
kME in WT
kME in Trem2 KO
1.0
0.5
0.0
−0.5
−1.0
−1.0
−0.5
0.0
0.5
1.0

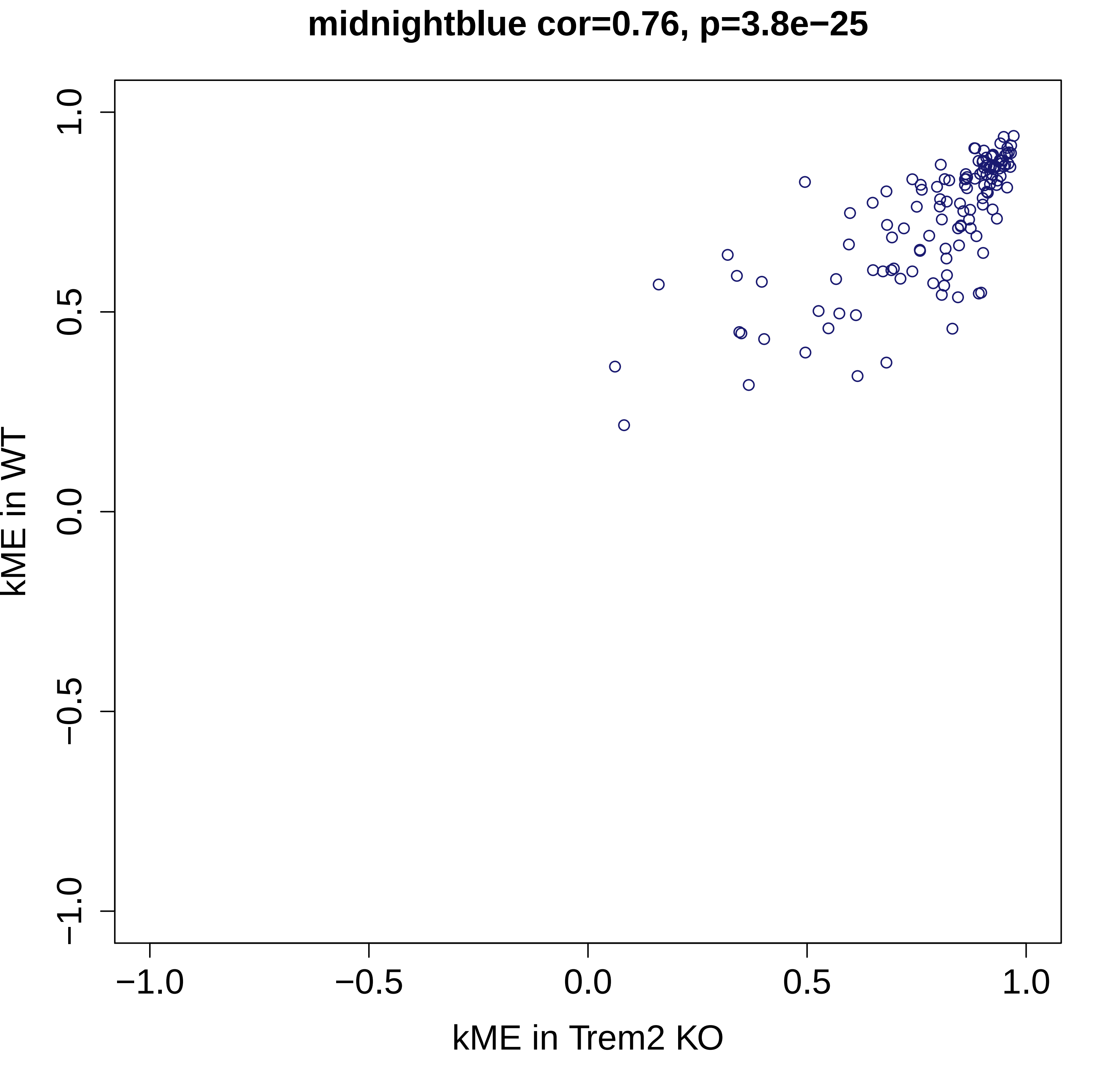
midnightblue cor=0.76, p=3.8e−25
kME in WT
kME in Trem2 KO
1.0
0.5
0.0
−0.5
−1.0
−1.0
−0.5
0.0
0.5
1.0
