# Supplementary material for: Loss of Trem2 in microglia leads to widespread disruption of cell coexpression networks in mouse brain
Source: Neurobiol Aging. 2018 Sep;69:151–66. doi: 10.1016/j.neurobiolaging.2018.04.019 (PMC6075941; doi:10.1016/j.neurobiolaging.2018.04.019)
Supplement: Supplementary file 18 [file mmc4.docx]

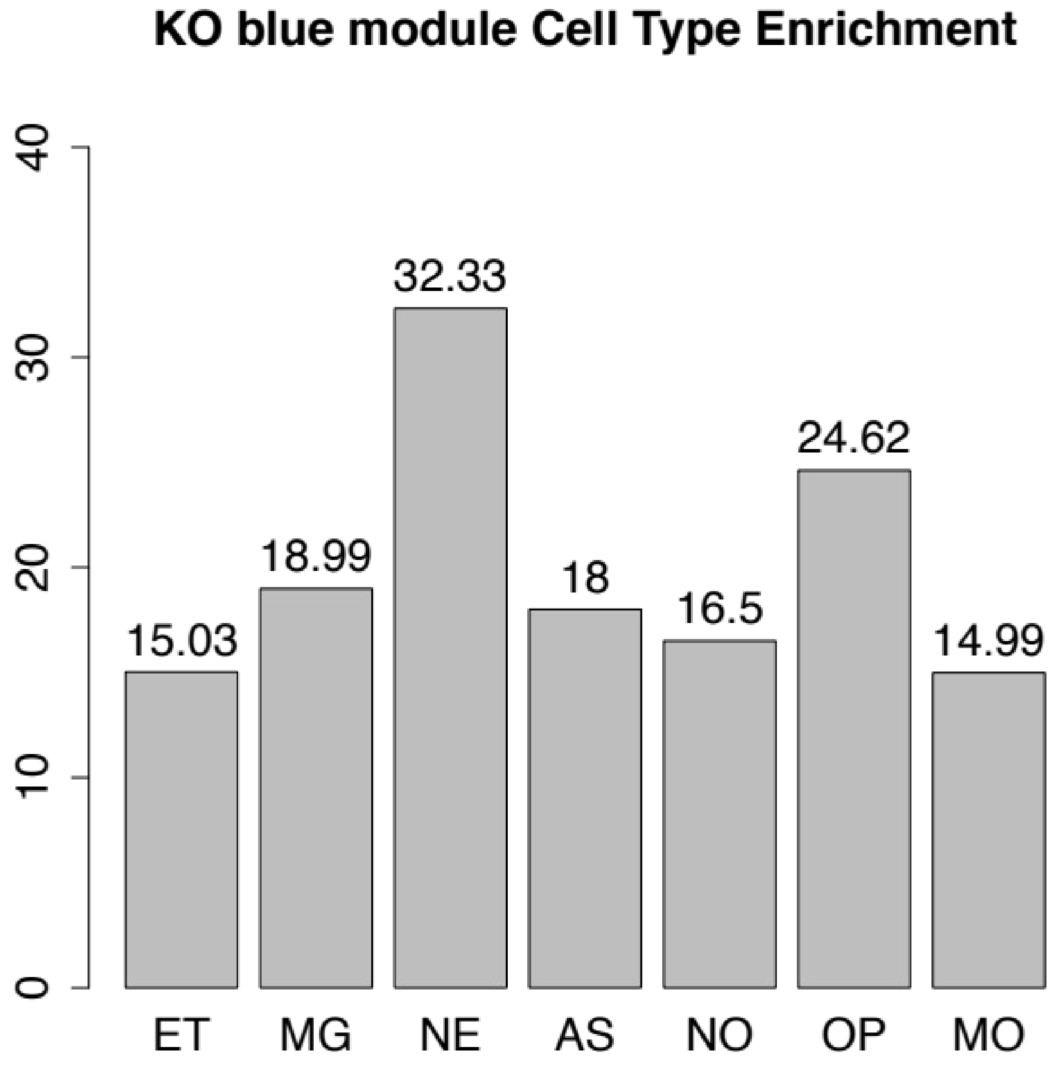

KO blue module Cell Type Enrichment
0
10
20
30
40
15.03
18.99
32.33
18
16.5
24.62
14.99
ET
MG
NE
AS
NO
OP
MO

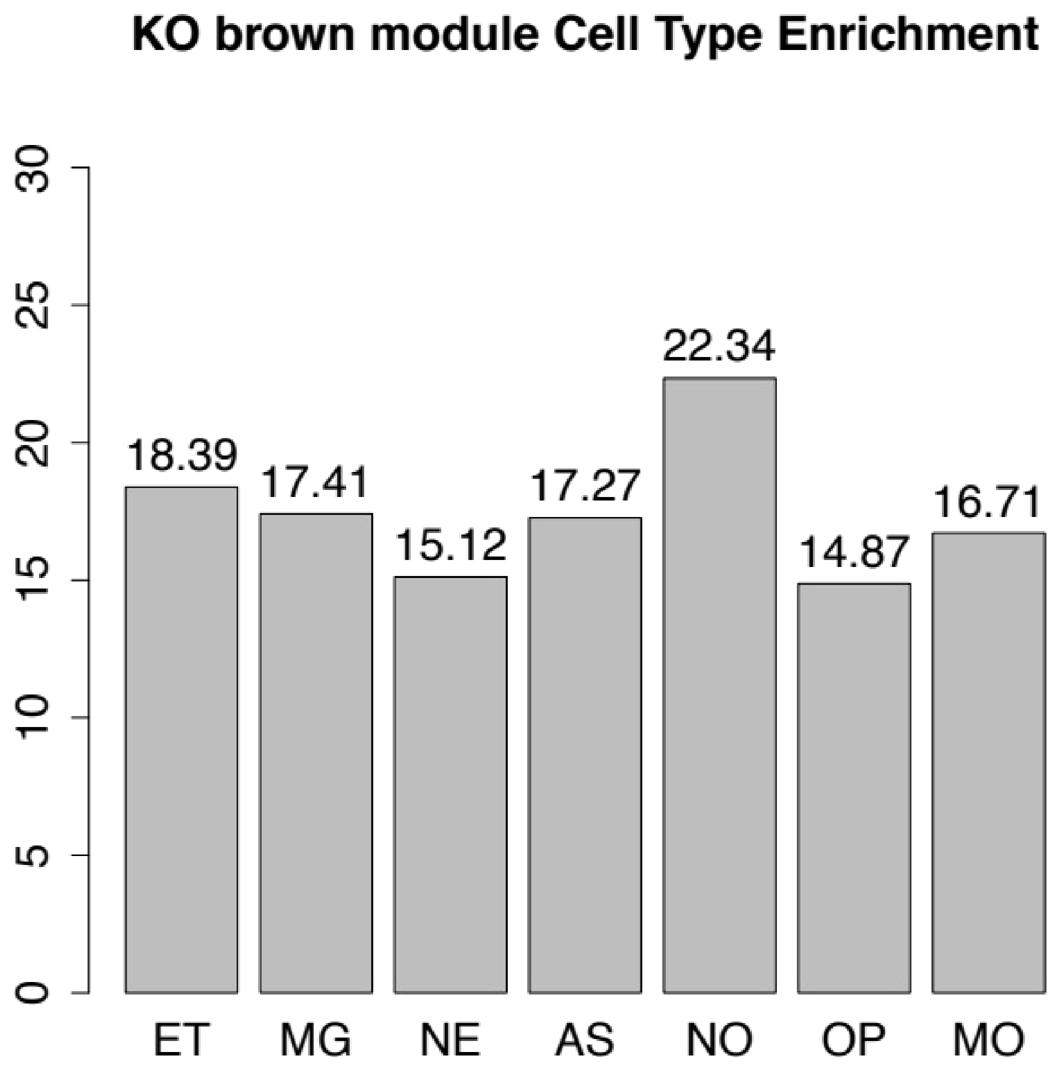
KO brown module Cell Type Enrichment
0
5
10
15
20
25
30
18.39
17.41
15.12
17.27
22.34
14.87
16.71
ET
MG
NE
AS
NO
OP
MO

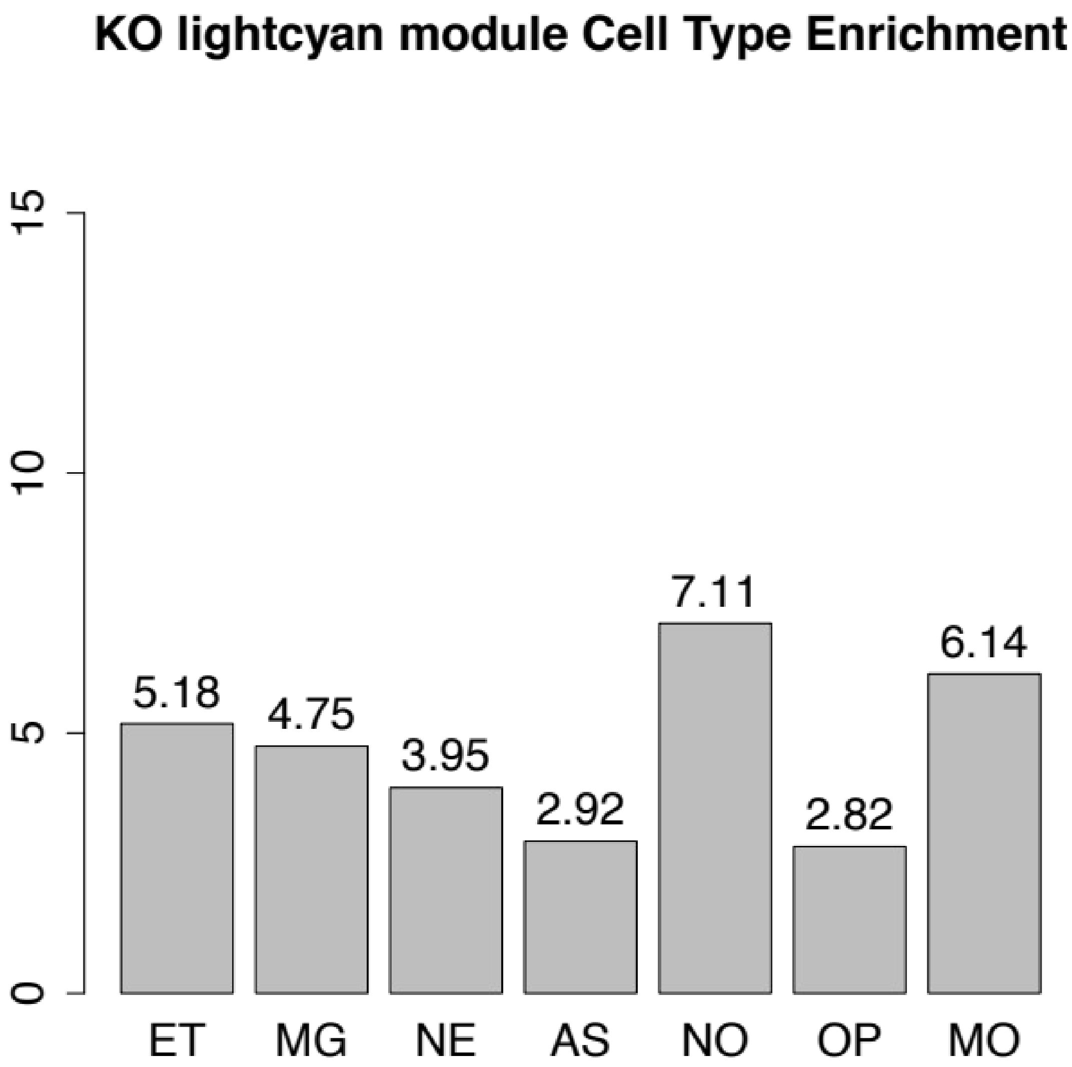
KO lightcyan module Cell Type Enrichment
15
10
5
0
5.18
4.75
3.95
2.92
7.11
2.82
6.14
ET
MG
NE
AS
NO
OP
MO

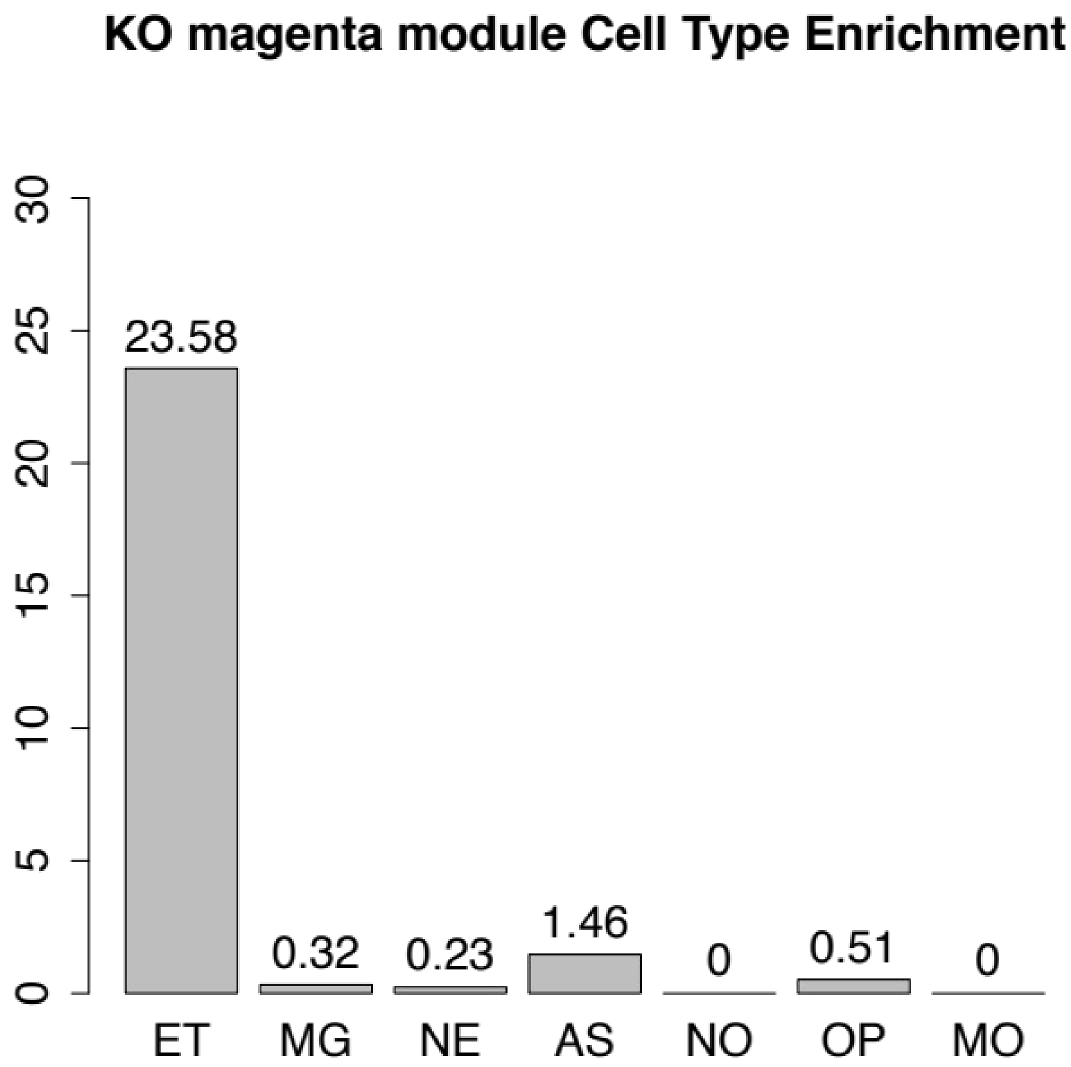

KO magenta module Cell Type Enrichment
30
25
20
15
10
5
0
23.58
0.32
0.23
1.46
0
0.51
0
ET
MG
NE
AS
NO
OP
MO

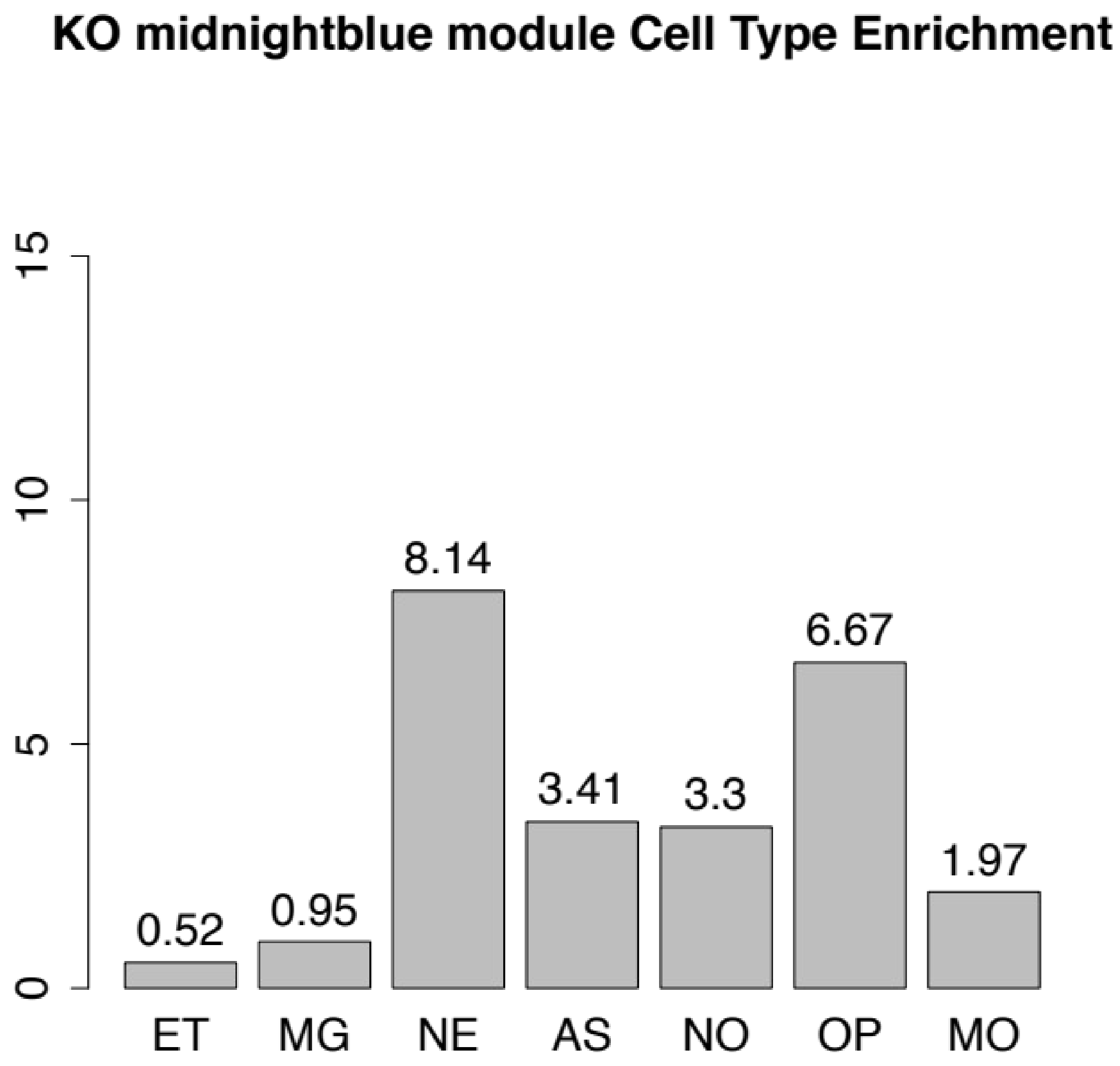
KO midnightblue module Cell Type Enrichment
0
5
10
15
0.52
0.95
8.14
3.41
3.3
6.67
1.97
ET
MG
NE
AS
NO
OP
MO

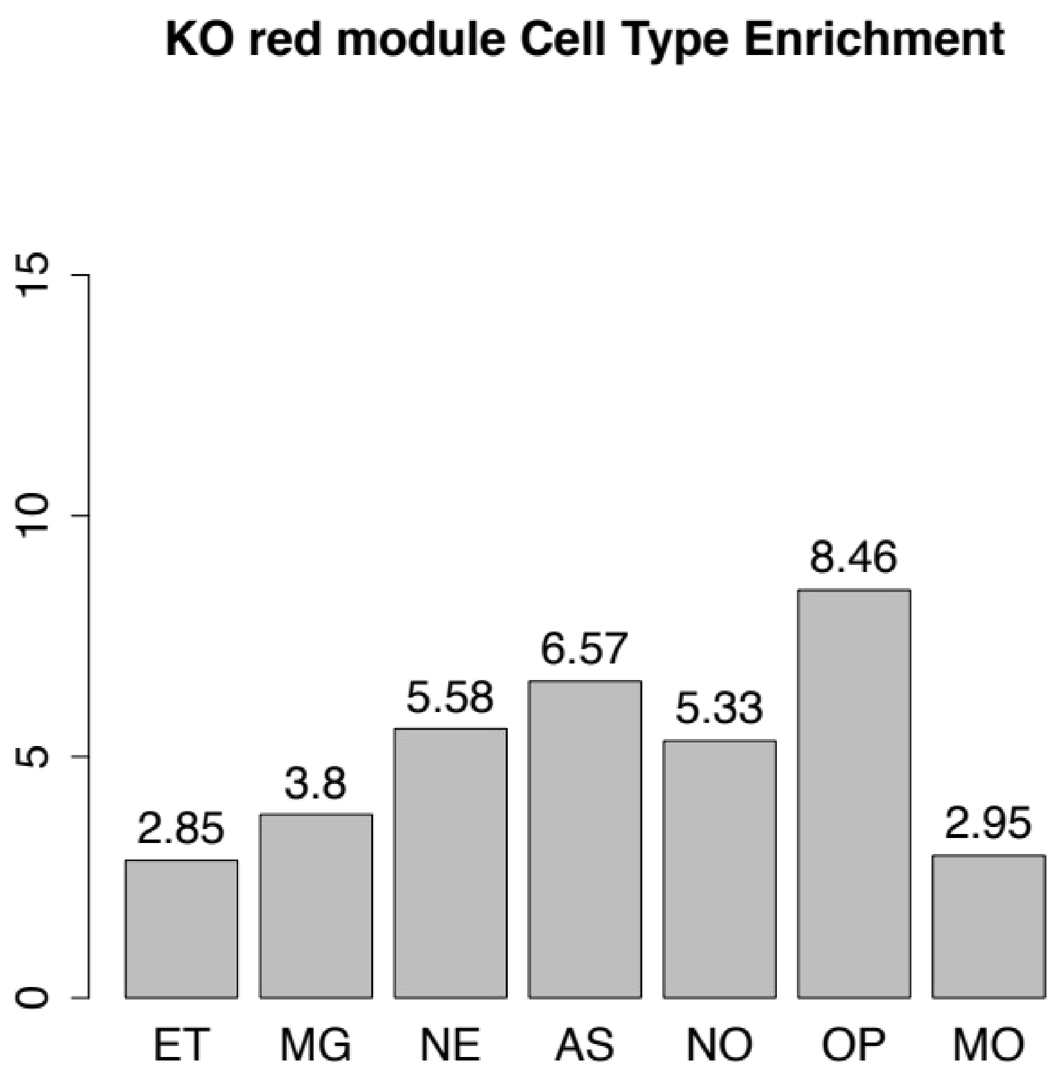
KO red module Cell Type Enrichment
15
10
5
0
2.85
3.8
5.58
6.57
5.33
8.46
2.95
ET
MG
NE
AS
NO
OP
MO

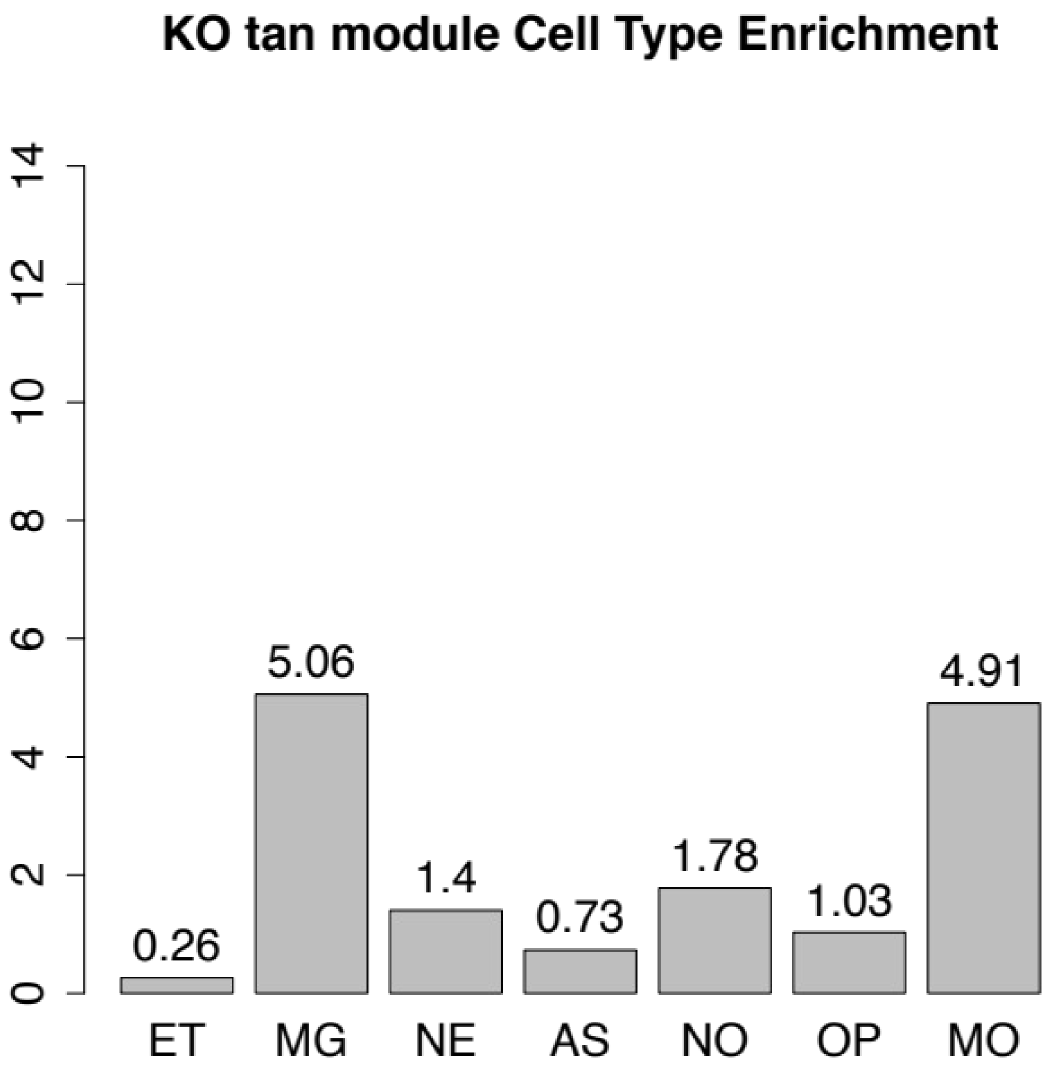
KO tan module Cell Type Enrichment
14
12
10
8
6
4
2
0
0.26
5.06
1.4
0.73
1.78
1.03
4.91
ET
MG
NE
AS
NO
OP
MO

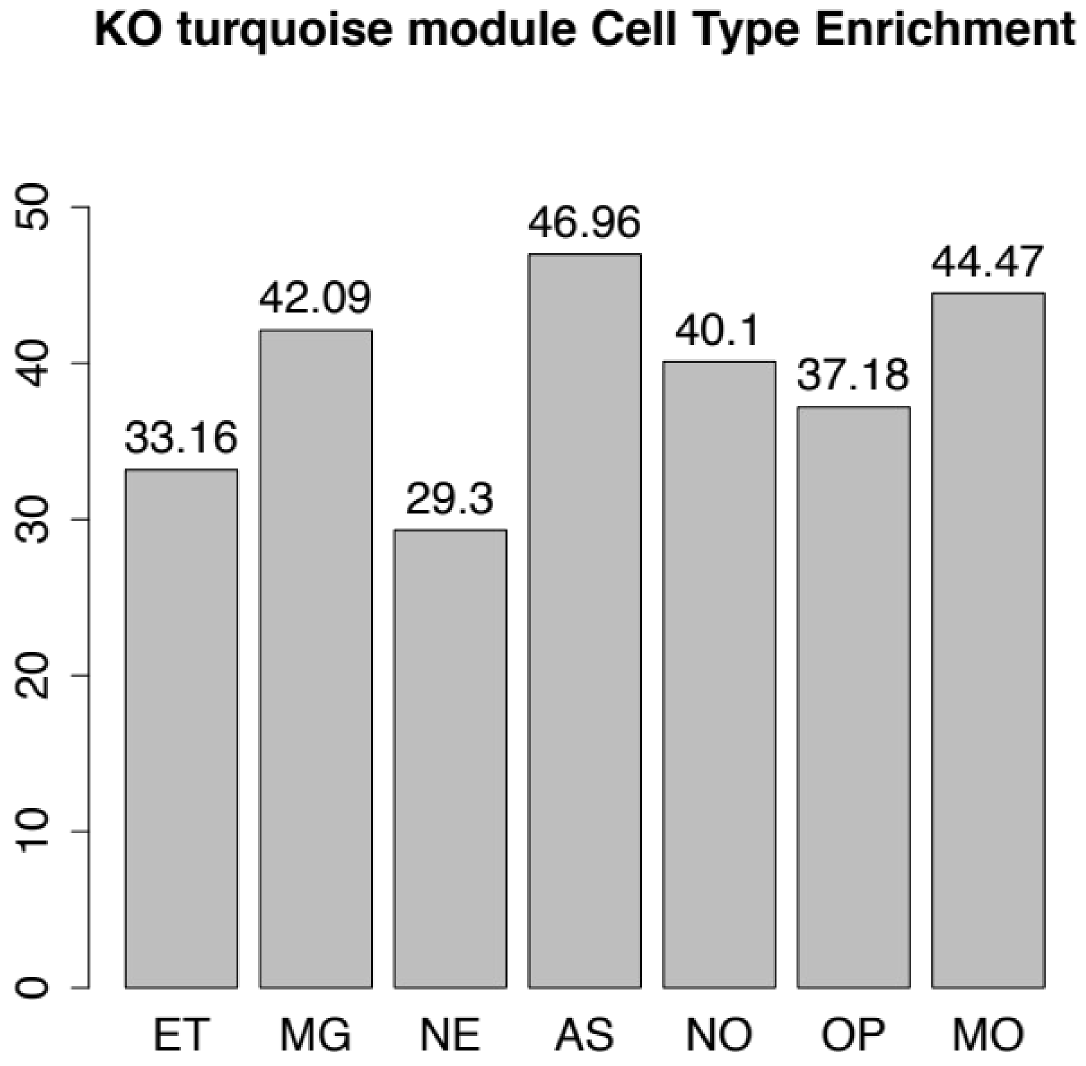
KO turquoise module Cell Type Enrichment
50
40
30
20
10
0
33.16
42.09
29.3
46.96
40.1
37.18
44.47
ET
MG
NE
AS
NO
OP
MO

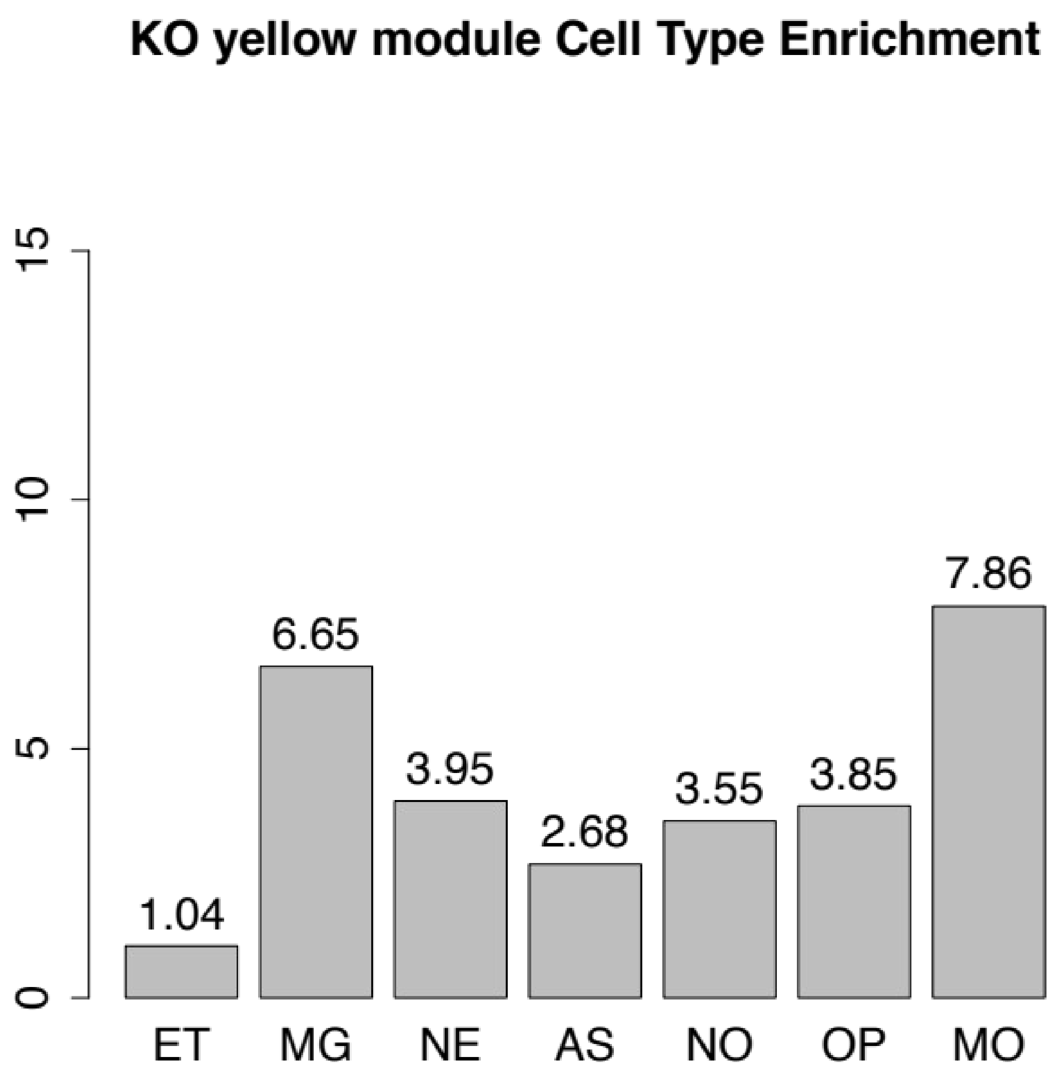
KO yellow module Cell Type Enrichment
15
10
5
0
1.04
6.65
3.95
2.68
3.55
3.85
7.86
ET
MG
NE
AS
NO
OP
MO

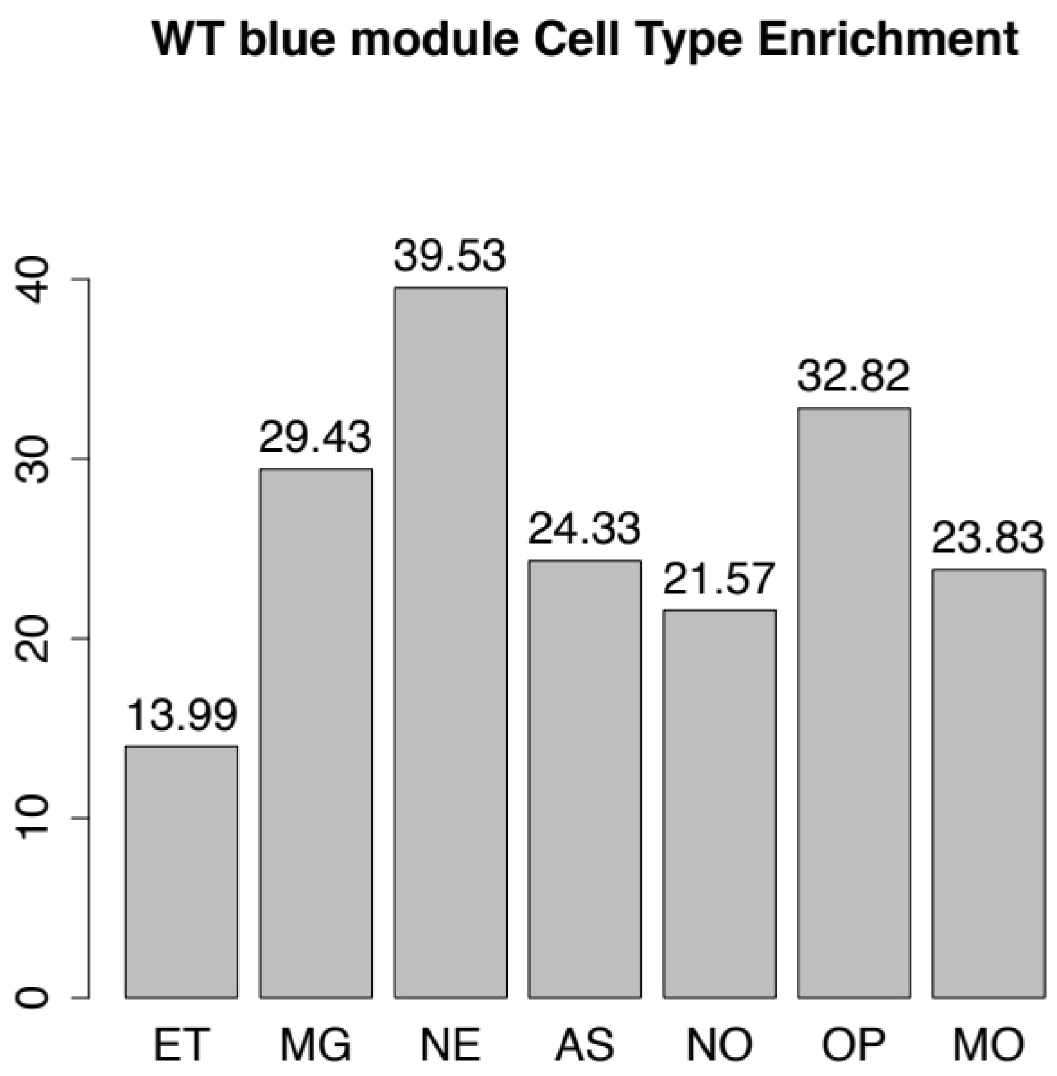
WT blue module Cell Type Enrichment
0
10
20
30
40
13.99
29.43
39.53
24.33
21.57
32.82
23.83
ET
MG
NE
AS
NO
OP
MO

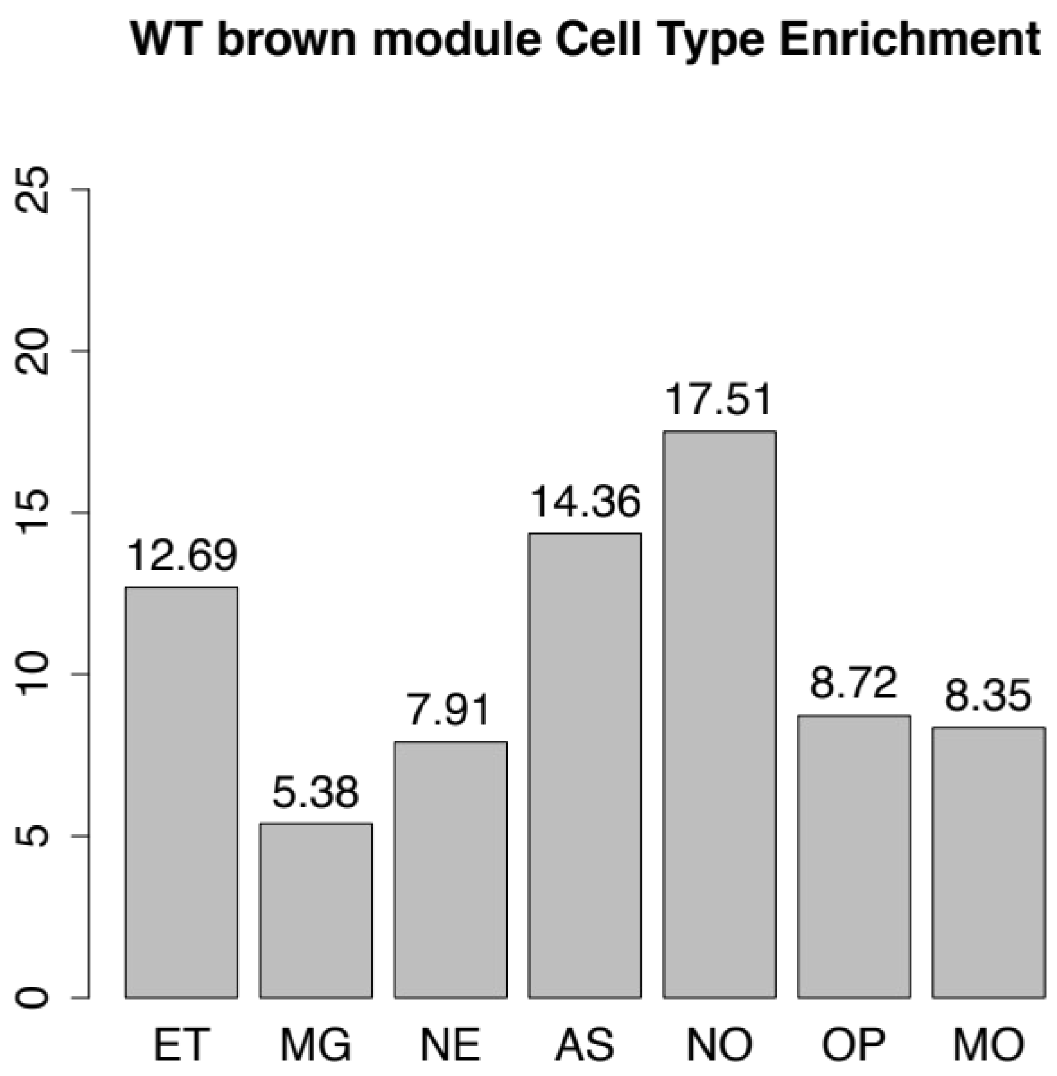
WT brown module Cell Type Enrichment
25
20
15
10
5
0
12.69
5.38
7.91
14.36
17.51
8.72
8.35
ET
MG
NE
AS
NO
OP
MO

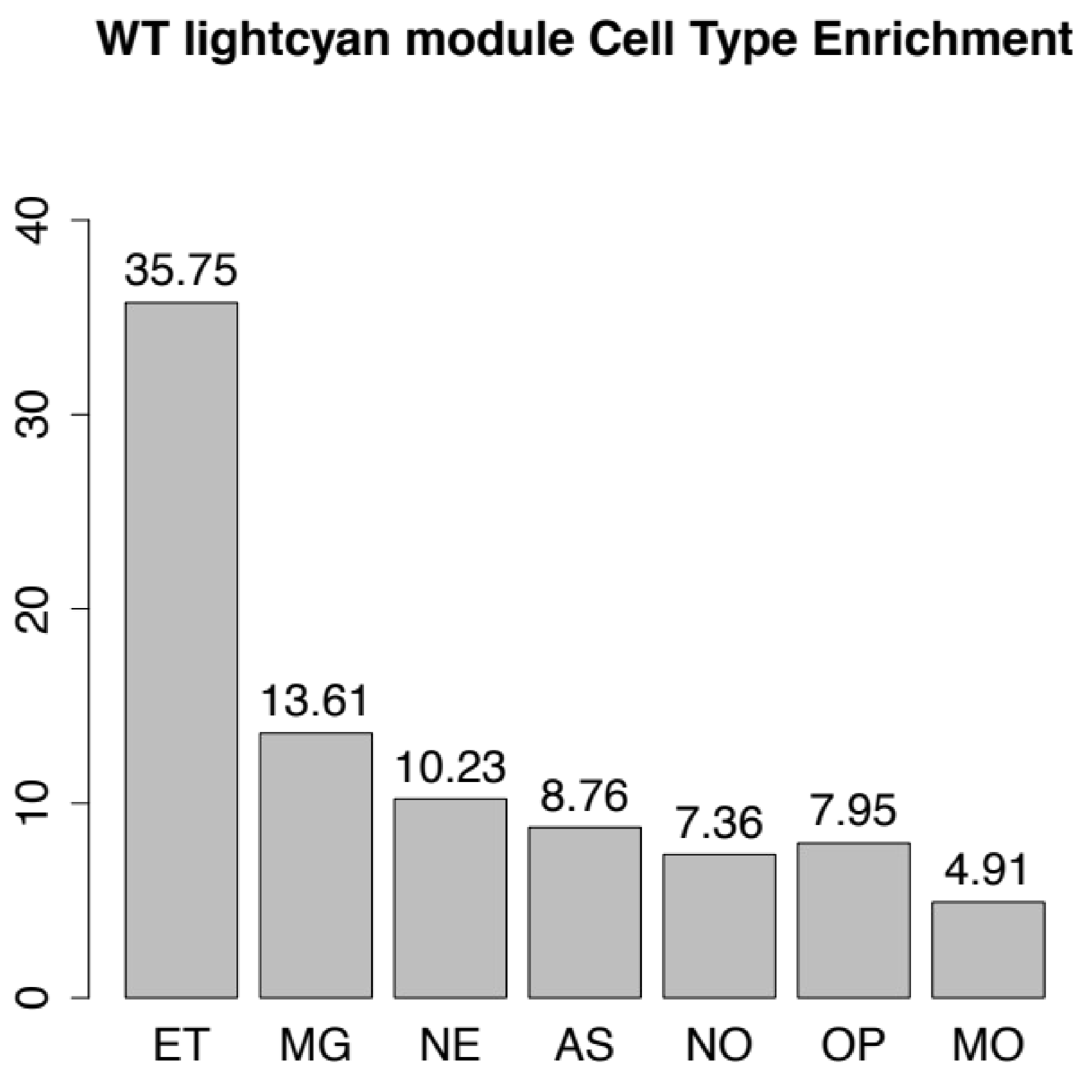

WT lightcyan module Cell Type Enrichment
40
30
20
10
0
35.75
13.61
10.23
8.76
7.36
7.95
4.91
ET
MG
NE
AS
NO
OP
MO

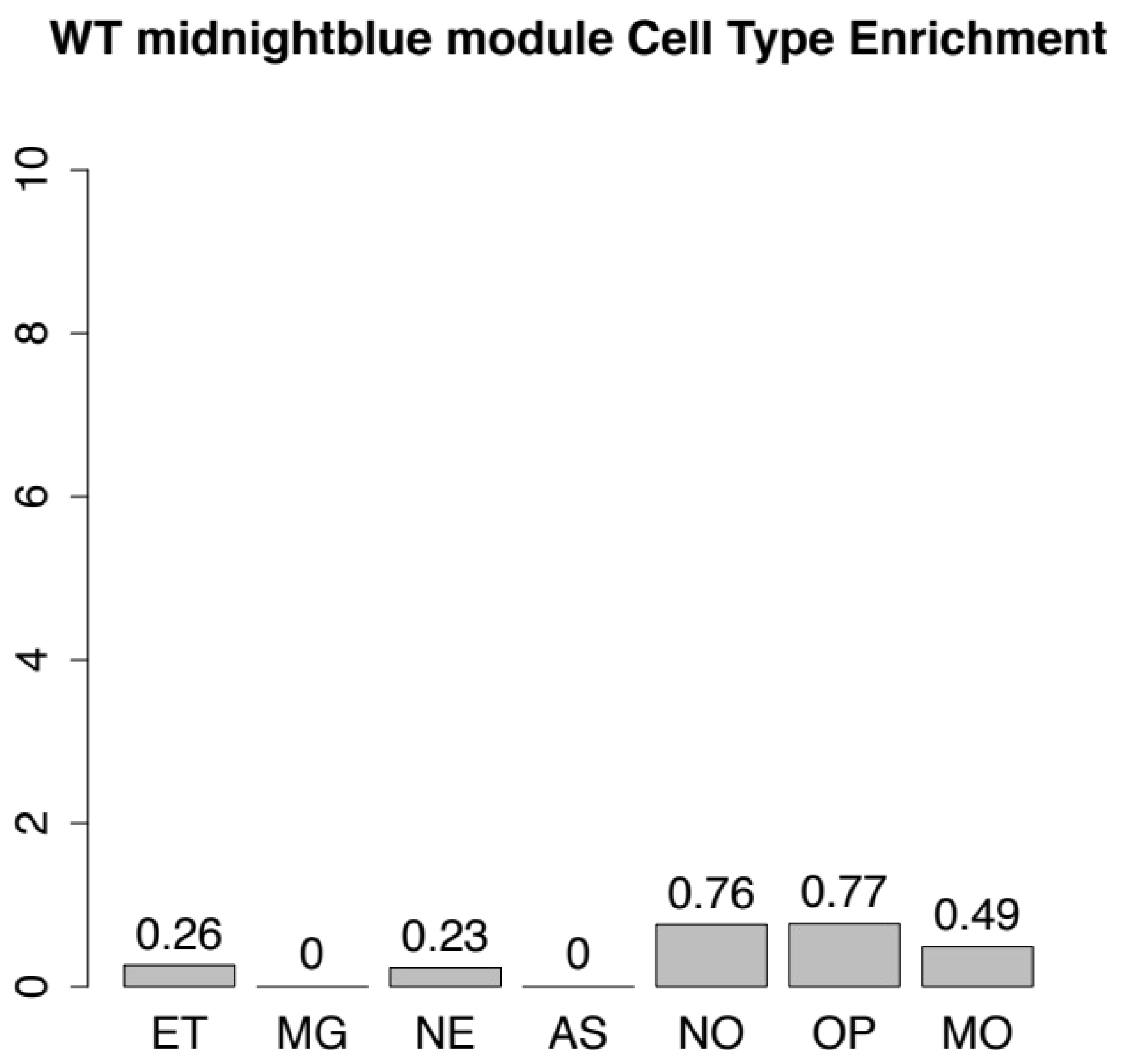
WT midnightblue module Cell Type Enrichment
0
2
4
6
8
10
0.26
0
0.23
0
0.76
0.77
0.49
ET
MG
NE
AS
NO
OP
MO

WT pink module Cell Type Enrichment

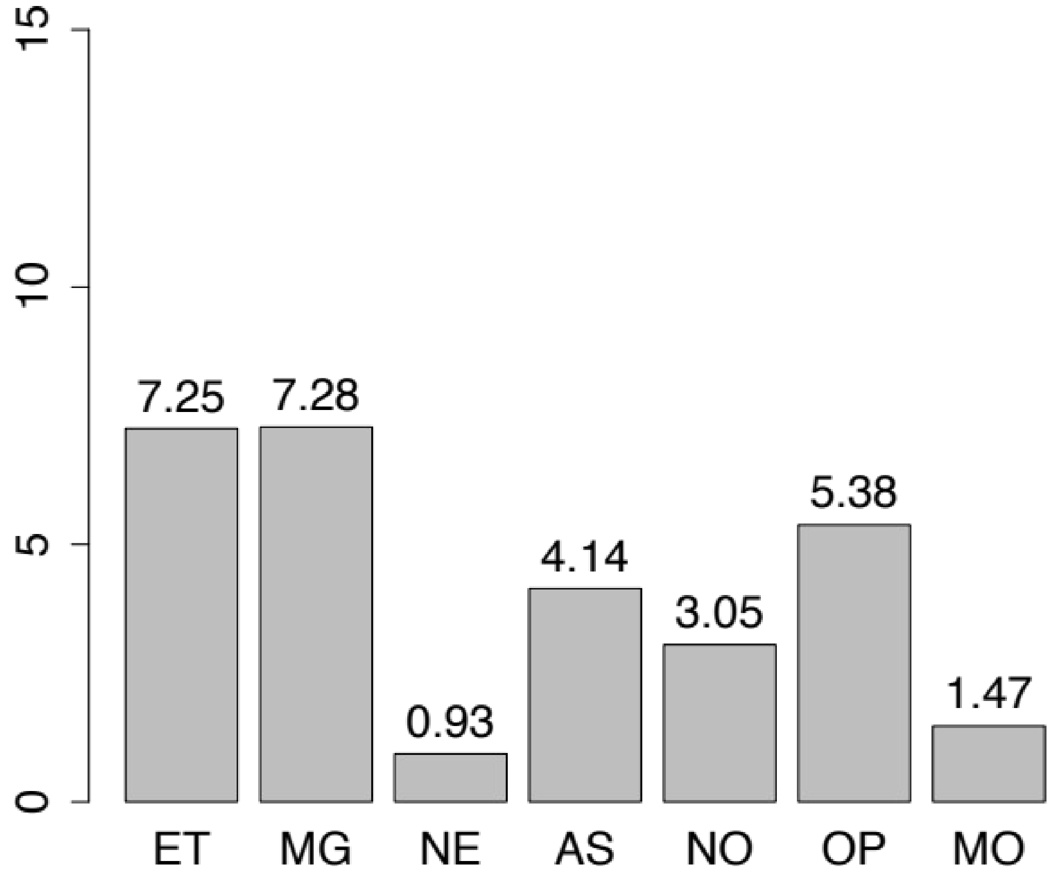

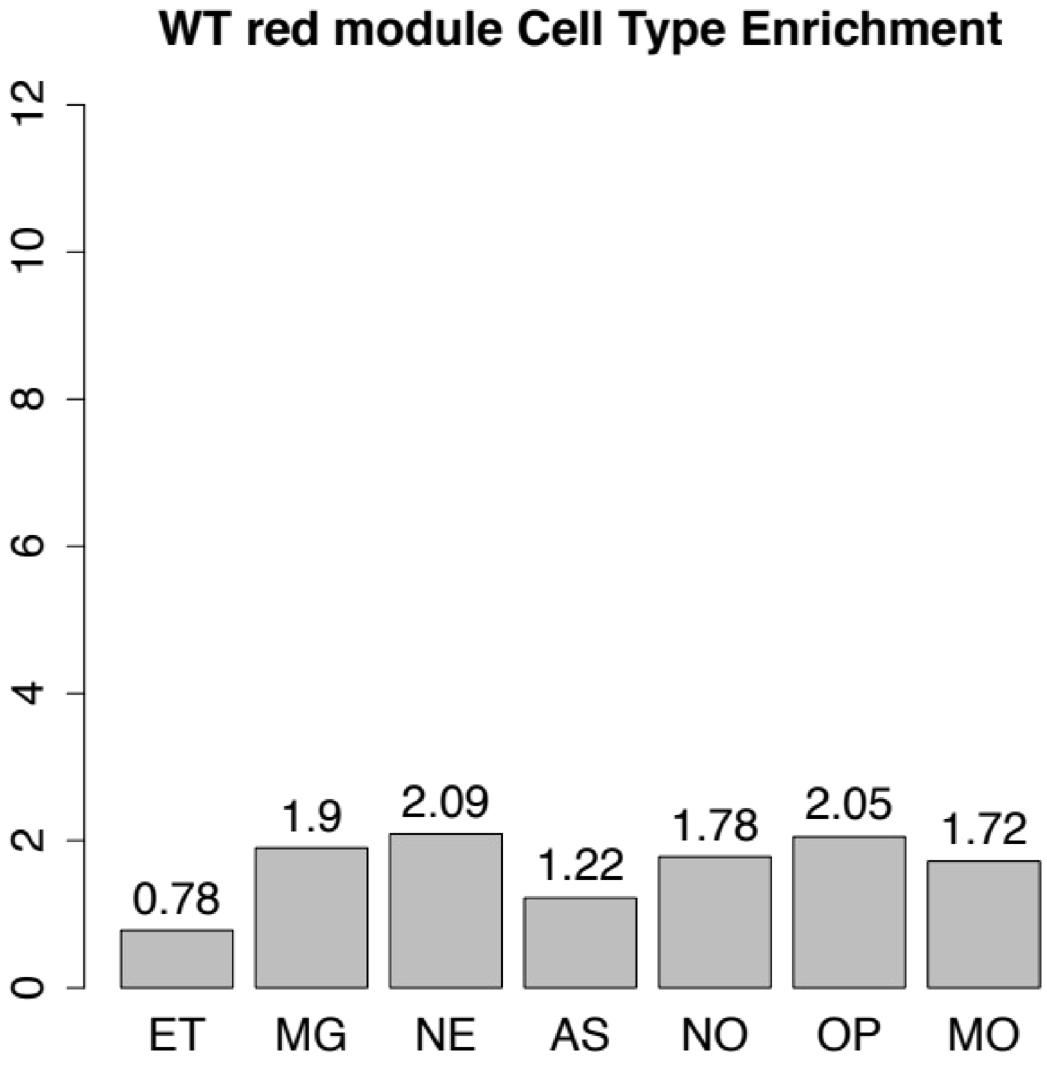

WT red module Cell Type Enrichment
0
2
4
6
8
10
12
0.78
1.9
2.09
1.22
1.78
2.05
1.72
ET
MG
NE
AS
NO
OP
MO

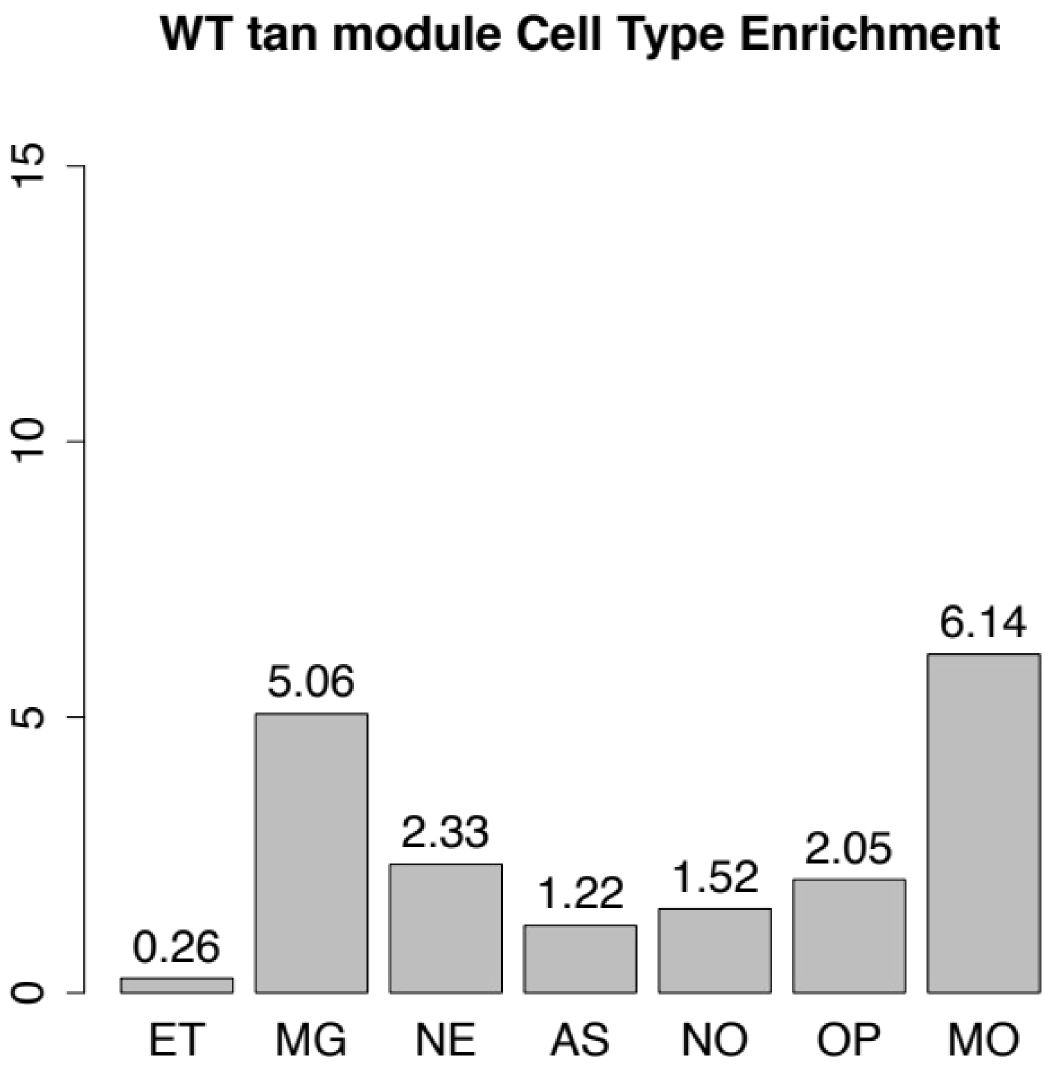
WT tan module Cell Type Enrichment
15
10
5
0
0.26
5.06
2.33
1.22
1.52
2.05
6.14
ET
MG
NE
AS
NO
OP
MO

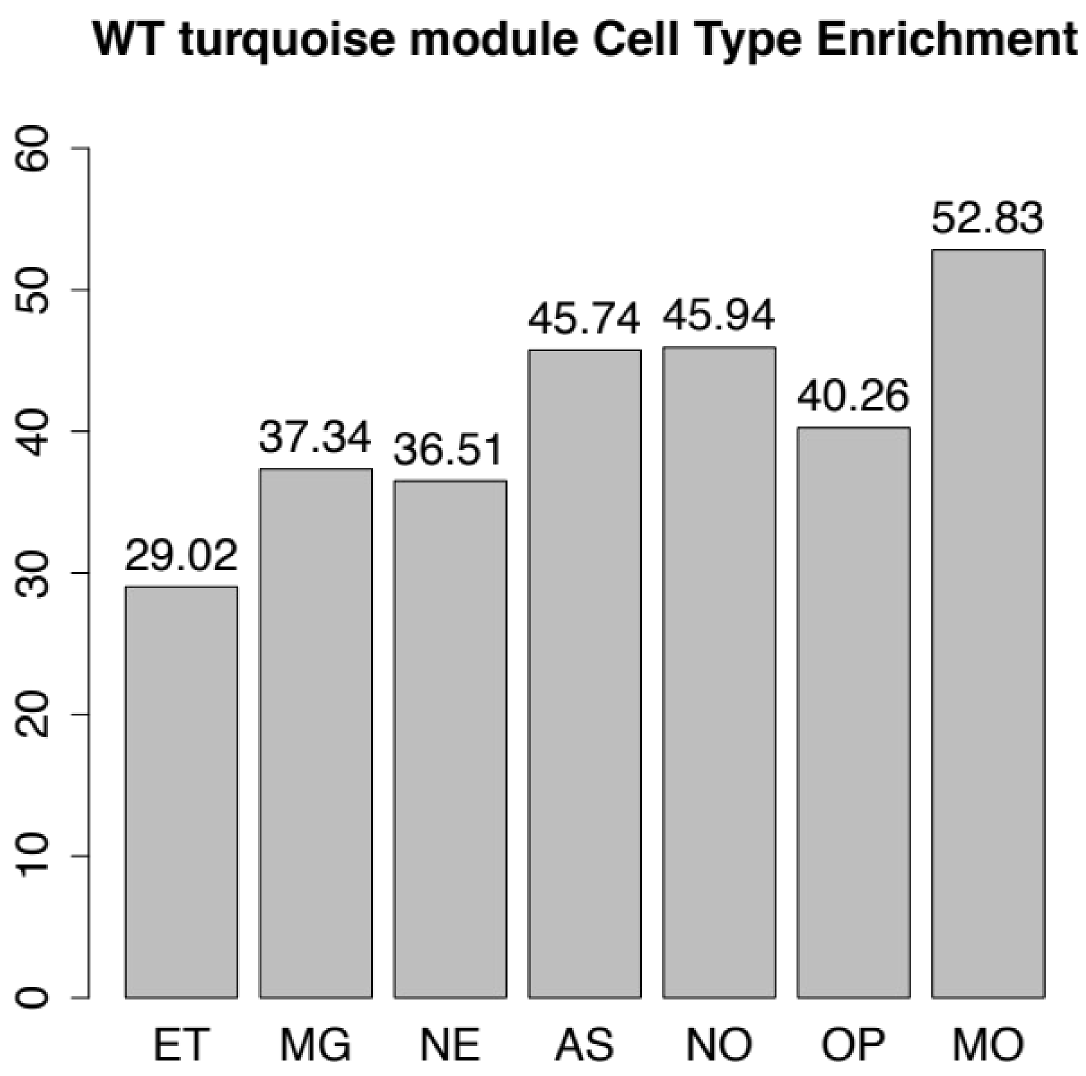
WT turquoise module Cell Type Enrichment
0
10
20
30
40
50
60
29.02
37.34
36.51
45.74
45.94
40.26
52.83
ET
MG
NE
AS
NO
OP
MO
